# Cortical Morphometric Similarity Remodeling in Traumatic Brain Injury Links Cognitive Impairments with Transcriptional Changes and Type‐Specific Cells

**DOI:** 10.1002/advs.202415262

**Published:** 2025-02-07

**Authors:** Yizhen Pan, Zhuonan Wang, Xiang Zhang, Wenpu Zhao, Haonan Zhang, Xuan Li, Xiaoyan Jia, Qiuyu Ji, Bo Yin, Guanghui Bai, Tingting Wu, Zhiqi Lee, Jierui Ding, Lei Shi, Jie Zhang, David H. Salat, Lijun Bai

**Affiliations:** ^1^ Department of Biomedical Engineering School of Life Science and Technology The Key Laboratory of Biomedical Information Engineering Ministry of Education Xi'an Jiaotong University Xi'an 710049 China; ^2^ PET‐CT Center The First Affiliated Hospital of Xi'an Jiaotong University Xi'an 710061 China; ^3^ Department of Neurosurgery The Second Affiliated Hospital and Yuying Children's Hospital of Wenzhou Medical University Wenzhou 325000 China; ^4^ Department of Radiology The Second Affiliated Hospital and Yuying Children's Hospital of Wenzhou Medical University Wenzhou 325000 China; ^5^ Department of Clinical Laboratory Shuguang Hospital Affiliated to Shanghai University of Chinese Traditional Medicine Shanghai 201203 China; ^6^ Department of Radiation Medicine School of Preventive Medicine Air Force Medical University Xi'an 710032 China; ^7^ Athinoula A. Martinos Center for Biomedical Imaging Department of Radiology Massachusetts General Hospital Charlestown MA 02114 USA

**Keywords:** cells, cortical structural abnormality, morphometric similarity network, transcription, traumatic brain injury

## Abstract

The heterogeneous injuries and resulting cognitive deficits pose significant challenges in the clinical management of mild traumatic brain injury (mTBI). However, the pathophysiological mechanisms related to heterogeneities of mTBI are still unclear. This study aims to explore the mechanisms underlying brain remodeling by examining the morphometric similarity (MS) alterations and corresponding transcriptomic signatures across adult and pediatric mTBI (adult mTBI: 112 acute patients, 47 follow‐up chronic patients, 66 healthy controls [HCs]; pediatric mTBI: 30 acute patients, 31 HCs). A healthy adult cohort (N = 840) is included to derive the modularized brain MS networks representing interregional cortical connectivity. Subsequently, cortical MS remodeling patterns are identified involving mostly MS increases in the frontal modules with typical high MS and decreases in the occipital module with typical low MS, with more pronounced changes observed in the developing brain with mTBI. The abnormal MS changes are correlated with variable cognitive impairments. Moreover, cortical MS remodeling is also associated with the genes enriched in CA1 pyramidal cells and neuron‐specific biological processes. The transcription‐related cortical remodeling in mTBI might reveal the disruption of brain cellular architecture. Therapeutic modalities to intervene in specific cortex and tackle CA1 over‐activation might better encircle the neurobiology of TBI.

## Graphical abstract

A cross‐scale framework utilizing morphological similarity (MS) to connect macroscopic dysfunctions and microscopic changes after traumatic brain injury (TBI). The MS remodeling focused on frontal and occipital cortices is associated with heterogeneous cognitive outcomes after TBI and is spatially correlated with gene overexpression on CA1 pyramidal cells, providing insights into the pathophysiological mechanism and novel treatment targets in TBI.

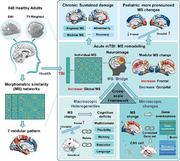

## 1 Introduction

Mild traumatic brain injury (mTBI) stands as one of the most prevalent neurological conditions characterized by the heterogeneity of the injuries sustained and the variability of the resulting cognitive deficits. The diverse clinical manifestations may arise from the heterogeneity of biological systems from the distinct neuroimaging pattern to the underlying variable microscopic changes.^[^ ^1^ ^]^ However, previous studies frequently concentrate on isolated scales, lacking a comprehensive, cross‐scale holistic perspective that bridges macro‐ and micro‐scales to investigate correlated abnormalities between brain alterations and pathophysiological processes.

It is widely acknowledged that ongoing cognitive difficulties following TBI relate to traumatic axonal injury, which disrupts the function of large‐scale networks that are crucial for cognitive processes. At the macroscale level, analysis of structural connectome using graph theory demonstrates that individuals with mTBI present a shift in small‐world network structure,^[^ ^2^ ^]^ characterized by longer characteristic path lengths^[^ ^3^ ^]^ and disconnection within network hubs.^[^ ^4^ ^]^ These alterations have been linked to cognitive impairments.^[^ ^3^, ^5^ ^]^ Although sufficient studies revealed the dysconnectivity of structural networks in mTBI, the current commonly used methods to construct structural connective networks including DTI‐based tractography and T1w‐based structural covariance analysis remain challenging. The tractography algorithm is particularly difficult to reliably identify long‐distance pathways with sparse connection strength,^[^ ^6^ ^]^ and the structural covariance network only uses a single morphological feature to establish a group‐level network.^[^ ^7^ ^]^ On this basis, morphometric similarity network (MSN) analysis represents a significant advancement in uncovering the macroscale cortical organization.^[^ ^8^ ^]^ MSN captures inter‐regional correlations of multiple morphometric features derived from multimodal MRI for each individual, rather than constructing a group‐level network based on a single feature. The combined analysis of multiple MRI morphometric features can also estimate the structural characteristics of the human cortex that are more closely associated with cytoarchitectonic classes. The biological validity of MSNs has been confirmed. Interconnected nodes within the MSN are likely to be axonally connected and exhibit similar cytoarchitectonic classes, as well as show high levels of co‐expressed genes.^[^ ^8^, ^9^ ^]^ These findings were also supported by histological evidence from nonhuman primates.^[^ ^9b^ ^]^ As a reliable method, MSN has been applied to some brain disorders, such as schizophrenia, major depressive disorder (MDD), and Alzheimer's disease (AD).^[^ ^10^ ^]^ Given that mTBI often results in structural connection interruptions, the MSN method is suitable for evaluating the altered anatomical connectivity in mTBI patients.

The microscopic alternation underlying the brain's connectivity characteristics contributing to the behavioral/clinical outcomes remains elusive. At the microscale level, some indirect evidence suggested that TBI alters the expression of genes involved in immune, inflammatory, and glial function,^[^ ^11^ ^]^ triggering diverse transcriptional changes in brain tissues.^[^ ^11^, ^12^ ^]^ Particularly, microscopic cellular alterations, notably in terms of cellular composition, have been observed in both animal models and patients.^[^ ^13^ ^]^ TBI results in the excitotoxicity and release of damage‐associated molecular patterns.^[^ ^14^ ^]^ This often induces a profound immune response, which can persist for years post‐injury. However, the translation of these microscale mechanisms into the heterogeneous clinical manifestations seen in mTBI remains poorly understood. The Allen Human Brain Atlas (AHBA) transcriptional profiles provide a feasible approach to map gene expression to brain regions, thereby achieving the connection between neuroimaging and genes. A recent study combined MDD‐related morphological gradient changes to AHBA data, revealing the brain‐wide expression of genes enriched for neurobiologically relevant pathways.^[^ ^15^ ^]^ Further study is warranted to integrate neuroimaging data with gene transcripts, elucidating how microscale alterations resulting from mTBI contribute to macroscale brain dysfunctions.

Given the essential of neuroimaging in facilitating the crosstalk of macroscale and microscale, we used neuroimaging‐based MSN analysis to establish a cross‐scale, network‐based framework to link heterogeneous cognitive outcomes, brain morphological similarity (MS) remodeling, and genetic, cellular abnormalities in mild TBI. We hypothesize that morphological similarity remodeling in patients with mild TBI is associated with heterogeneous cognitive outcomes and is influenced by gene expression and specific cellular features. To verify this hypothesis, we first constructed the brain MSN modular parcellation pattern using 840 healthy individuals from the Human Connectome Project (HCP). Second, we identified abnormal morphometric similarity changes in both 112 adult mTBI (re‐examined at 6–12 months post‐injury follow‐up) and 30 pediatric mTBI patients, associated with cognitive deficits of varying severity. We then related such macroscale remodeling with disrupted cellular circuits using brain‐specific gene expressions provided by the AHBA.

## 2 Results

### 2.1 Experimental Design

**Figure 1:**
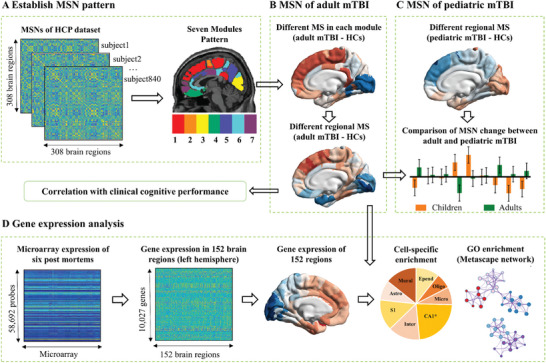
Schematic overview of data analysis. A) Establish MSN pattern. The seven modules pattern was partitioned from the morphological similarity network (MSN) on 840 healthy young participants. B) MSN of adult mTBI. The adult mTBI – HCs MSN differences across 7 modules and across 308 regions were calculated. C) MSN of pediatric mTBI. The pediatric mTBI – HCs MSN differences across regions and the comparison of MSN change between adult and pediatric patients were then computed. D) Gene expression analysis. The microarray expression of six postmortems from the Allen Human Brain Atlas (AHBA) was assigned to 152 brain regions of the left hemisphere. The regional gene expressions of adult mTBI patients were then obtained to identify the gene sets most associated with mTBI‐related MSN change. The cell‐specific enrichment and GO enrichment were performed on the selected gene sets. MSNs, morphological similarity networks; HCP, Human Connectome Project; MS, morphological similarity; T1w, T1 weighted; GO, gene ontology.

The current study combined multimodal MRI imaging data, neuropsychological assessments, and gene transcriptional profiles to reveal the associated cross‐scale changes in patients with mTBI (**Figure** **1**). We included three independent cohorts: healthy young adult dataset curated by HCP,^[^ ^16^ ^]^ adult and pediatric mTBI datasets as well as their matched healthy controls (HCs). Motivated by a recent finding that first defined the morphometric similarity network (MSN) mapping and suggested four modules of the topological organization in 300 adolescents and young adults aged 14–24 years,^[^ ^8^ ^]^ we constructed the MSN modular parcellation pattern in the adult population using the relatively larger sample of 840 healthy individuals from the HCP datasets. Then, adult mTBI cohort (N = 112) was used to examine the case‐control changes in MSN modules and transcriptional enrichment pathways. We also included the pediatric mTBI cohort (N = 30) to validate the altered MSN‐related pattern in the developing brain with mTBI exposure. There were no significant demographic differences between patients and matched HCs in both adult and pediatric mTBI cohorts. For the adult mTBI cohort, patients received neurobiological test and exhibited worse cognitive performances (Supporting Information S01, Table S1 and S2).

### 2.2 Specific Brain Modules Divided by MSN

**Figure 2:**
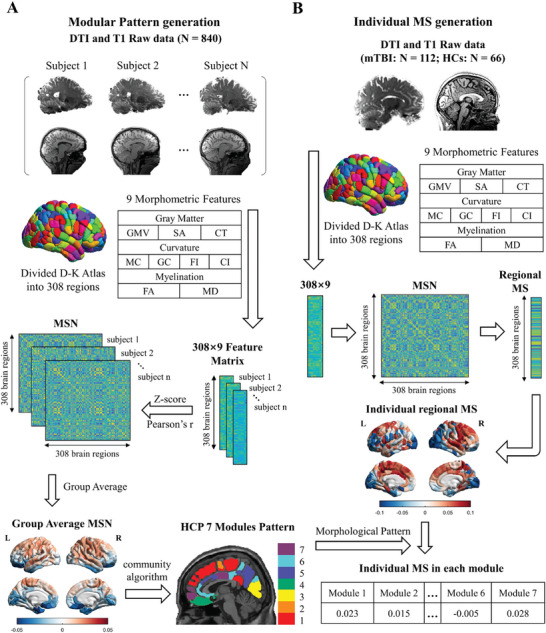
Schematic of the group‐level pattern and individualized MSN construction approach. A) The group pattern of modularity based on MSN was generated using HCP dataset containing 840 healthy subjects. The brain was parcellated into 308 cortical regions using DKT‐308 atlas (a subdivision of the Desikan–Killiany atlas). Nine morphological features including gray matter features, curvature features, and myelination features were extracted from T1w and DTI data of each subject to generate the Z‐score normalized 308 × 9 feature matrix. Each MSN with the dimension of 308 × 308 was obtained by calculating Pearson's correlation coefficient between each pair of cortical regions. Louvain community modularity algorithm was used on group average MSN to construct seven modules pattern. B) The individual MSN was obtained using the same procedure described above for group‐level MSN. The individual nodal regional MS with the dimension of 1 × 308 was obtained by measuring the mean value of each row (or column) of MSN except self‐connection, which represents the similarity of the selected region with other regions in the brain. Each of the 308 regions was then assigned to one of the seven modules to calculate the MS in each module. T1w, T1‐weighted; GMV, gray matter volume; SA, surface area; CT, cortical thickness; MC, mean curvature; GC, gaussian curvature; FI, folding index; CI, curvature index; FA, fractional anisotropy; MD, mean diffusivity; MSN, morphological similarity network; HCP, Human Connectome Project; MS, morphological similarity.

We began by generating the MSN modular parcellation patterning using the large sample of the HCP dataset (N = 840) (**Figure** **2**A). Specifically, we computed the MSN from the interregional Pearson's correlation of nine morphometric features derived from T1‐weighted (T1w) and diffusion‐weighted imaging (DWI) images acquired from each participant. We then used the Louvain modularity algorithm to partition closely connected nodes in the group‐level MSN into the same module to obtain seven spatially continuous morphological modules (Figure 2A). Different network sparsity and variable values of the parameter of the Louvain modularity algorithm were used to verify the robustness of the seven‐module parcellation pattern (Supporting Information S02, Figure S1).

The robust 7‐module solution was basically aligned with the macrostructure. The regions in module 1, module 4, and module 7 were mainly located in the frontal lobe. Module 2 and module 3 regions were separately concentrated in the insula and occipital lobe. Module 5 and module 6 regions are mainly located in the parietal and temporal lobes. Compared to the modular structure of MSNs derived from adolescents,^[^ ^8^ ^]^ the modules of adults exhibited significant regional differentiations and specializations. The frontal lobe was further subdivided into the orbitofrontal cortex (module 4) and remained parts of the frontal regions (module 1 and module 7). The insula was extracted and separated as an independent module. The regional MS was then calculated as the mean value of each row (or column) of MSN (without self‐connection). At the group level, regional MS varied greatly across different modules, with a higher value in the frontal cortices (module 1 and 7), a lower value in the insular, occipital, and orbital frontal cortices (module 2, 3, and 4), and a middle value in the parietal and temporal lobe (Supporting Information S03, Figure S2). This gradient was anchored at two extreme ends by the frontal with more similar morphometric profiles, and the occipital, insula, and orbital frontal cortices with higher degrees of histological differentiation.

**Figure 3:**
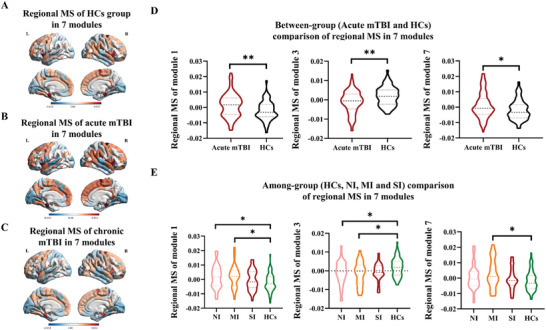
MSN changes in morphological modules in patients with mTBI. A–C) show the mean regional MS in the seven morphological modules in HCs group, acute mTBI patients, and the follow‐up chronic mTBI patients. D) Comparison between acute mTBI and HCs in the three modules with significant different regional MS. E) Among‐group comparison of modularized regional MS in HCs and patients of three cognitive subgroups (NI, MI, and SI subgroups) in the acute phase. The * indicates the significant difference with FDR‐corrected *p*‐value between 0.01 and 0.05 and the ** displays the FDR‐corrected *p*‐value between 0.001 and 0.01. MS, morphological similarity; NI, no impairment; MI, moderate impairment; SI, severe impairment.

The individual MS at each region was also calculated. Each brain region (defined by the fine‐grained DKT‐308 atlas^[^ ^17^ ^]^) was assigned to one of the seven modules and the mean MS for each module was then obtained (Figure 2B). For each module, the mean regional MS for each group (**Figure** **3**A–C) and between‐group comparison (Figure 3D,E) were conducted for the 66 HCs, 112 acute mTBI patients and 47 chronic mTBI patients. In HCs group, module 1, 2, and 7 had higher regional MS, while module 3 and 4 had lower regional MS. Although age distribution varied between HCs group and HCP dataset, the distribution pattern of regional MS in the main modules remained stable for both HCs and HCP. Compared with HCs, individuals with acute mTBI exhibited significantly decreased regional MS in the occipital module (module 3: *p* = 0.002, FDR corrected), and increased regional MS in the frontal modules (module 1: *p* = 0.008, FDR corrected; module 7: *p* = 0.035, FDR corrected) (Figure 3D). However, no significant differences in regional MS were found between the chronic mTBI and HCs.

Neuropsychological reports were reviewed to rate each patient's degree of cognitive impairments across different domains. Each patient's status was rated in four cognitive domains: cognitive information processing speed, executive function, working memory, and language ability (details in the Experimental Section). Then, we divided the mTBI patients into three subgroups, such as no‐domain impairment (NI), moderate impairment (MI) with single‐domain impairment, and severe impairment (SI) with multi‐domain impairments defined in our previous study.^[^ ^18^ ^]^ Only for patients with no or single domain of cognitive impairment, regional MS increased in the frontal modules (module 1 and 7) and decreased in the occipital module (module 3) (all for *p* < 0.05, FDR corrected) (Figure 3E). Patients with multiple domains of impairment presented the same level of MS in all modules compared to HCs.

### 2.3 MTBI‐Related Changes in Regional MSN

**Figure 4:**
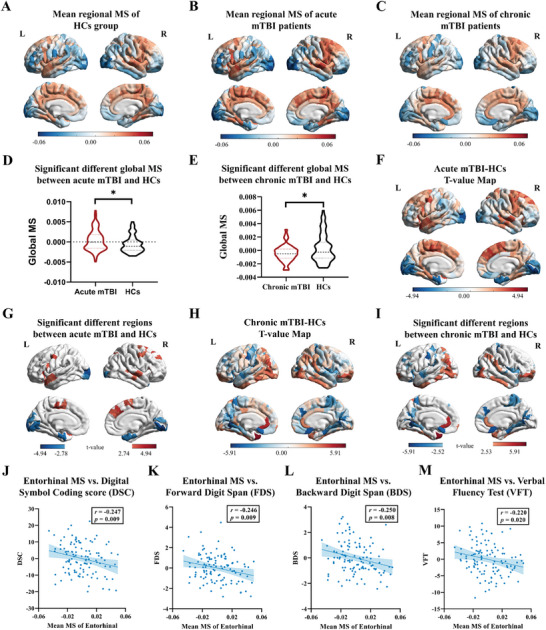
Differences in global MS and regional MS between adult mTBI patients and HCs. A–C) display the mean regional MS of HCs group, acute mTBI patients, and follow‐up chronic mTBI patients. D,E) show the between‐group differences of global MS. F) shows the case (acute mTBI)‐control (HCs) t‐value map of reginal MS. G) shows the regions with significantly altered reginal MS in case (acute mTBI)‐control (HCs) t‐value map (*p* < 0.05, FDR corrected). H) shows the case (chronic mTBI)‐control (HCs) t‐value map of reginal MS. I) shows the regions with significantly altered reginal MS in case (chronic mTBI)‐control (HCs) t‐value map (*p* < 0.05, FDR corrected). J–M) show the significant negative correlation between MS of the entorhinal cortex and multi‐dimensional cognitive performances. The * indicates the significant difference with *p* value between 0.01 and 0.05. MS, morphological similarity; DSC, Digital Symbol Coding score; FDS, Forward Digit Span; BDS, Back Digit Span; VFT, Verbal Fluency Test.

The regional MS of both HCs and mTBI were shown in the **Figure** **4**A–C (ranging from −0.05 to 0.04 in HCs, from −0.06 to 0.04 in the acute mTBI and from −0.04 to 0.03 in the chronic mTBI). Globally, the MS increased during the acute phase of mTBI compared to HCs (*p* = 0.018), while decreased in 6–12 months post‐injury (*p* = 0.039) (Figure 4D,E).

Compared with HCs, individuals with acute mTBI exhibited bidirectional abnormal changes, for which increased MS mainly in the regions with more morphometric similarity and decreased MS particularly in the histologically differentiation regions (all for *p* < 0.05, FDR corrected). For instance, decreased regional MS were mainly located in the lateral occipital cortex and lingual gyrus within the module 3, while increased regional MS were mainly located in the superior frontal cortex and superior temporal cortex within the module 1 and 7 (Figure 4F,G, Supporting Information S04, Tables S3 and S4). A decreased regional MS in individuals with mTBI suggested the greater morphometric differentiation between this cortical region and the rest of the areas, can be interpreted as attenuated anatomical connectivity to and from the more differentiated cortical areas, and verse visa for increased regional MS. Though no significant difference of the MS at the module level, there was widely regional MS changes when these mTBI patients’ follow‐up at 6–12 months (all for *p* < 0.05, FDR corrected) (Figure 4H,I, Supporting Information S04, Tables S5 and S6).

To examine the relation between the abnormal MSN and cognitive performance, we calculated the correlation between the average MS in regions with the highest proportion of the altered MS and cognitive ratings (Supporting Information S05, Tables S7 and S8). We found significant correlation between the mean MS of the entorhinal cortex with multiple domains of cognitive scales, including Digital Symbol Coding score (DSC, *r* = −0.247, *p* = 0.009), Forward Digit Span (FDS, *r* = −0.246, *p* = 0.009), Back Digit Span (BDS, *r* = −0.250, *p* = 0.008), and Verbal Fluency Test (VFT, *r* = −0.220, *p* = 0.02) in acute mTBI (all survival after FDR corrected, Figure 4J–M). When follow‐up at 6–12 months post‐injury, there was only a marginal significant correlation between the mean MS of the inferior temporal cortex and the VFT (Supporting Information S05, Figure S3). Partial least squares (PLS) regression also indicated that the MS in the association cortex significantly correlated with the cognitive performance (Supporting Information S06, Figures S4 and S5 and Table S9).

**Figure 5:**
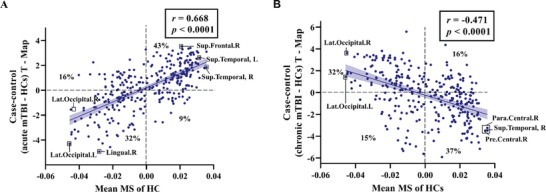
The correlation between mean MS of HCs and case‐control T‐Map. A) displays the correlation between mean MS of HCs and case‐control (acute mTBI – HCs) T‐Map. Most regions showed hypercoupling (43% of regions) or hyperdifferentiation (32%). B) displays the correlation between mean MS of HCs and case‐control (chronic mTBI – HCs) T‐Map. Most regions exhibited decoupling (37%) or dedifferentiation (32%). MS, morphological similarity.

The case‐control (mTBI – HCs) T‐value map (T‐Map) was obtained by calculating the two‐sided t‐statistic value of the between‐group comparison across every region. The significant spatial correlation was found between case‐control t‐map and the mean MS of HCs (*r*_(306)_ = 0.668, *p* < 0.0001 (**Figure** **5**A), inferring the larger case‐control differences in typically more connected regions. Positive case‐control t‐values and positive mean MS of HCs exhibited in 43% of regions, indicating the hypercoupling in individuals with acute mTBI relative to HC, while the negative t‐values and negative mean MS in 32% of regions suggested the hyperdifferentiation^[^ ^10b^ ^]^ in acute mTBI. In chronic mTBI, however, we found a completely opposite trend of MS changes. The case‐control t‐map was significantly negatively correlated with the mean MS of HCs (*r*_(306)_ = −0.471, *p* < 0.0001) (Figure 5B). Negative t‐values and positive mean MS exhibited in 37% of regions, indicating the decoupling in individuals with chronic mTBI relative to HC, while the positive t‐values and negative mean MS in 32% of regions suggested the dedifferentiation^[^ ^10b^ ^]^ in chronic mTBI. The detailed definition of hypercoupling, hyperdifferentiation, decoupling and dedifferentiation were also shown in Supporting Information S07 and Figure S6. Considering some of patients have not completed follow‐up investigation, we also repeated this finding in the 47 mTBI patients with both acute and follow‐up datasets (Supporting Information S08, Figure S7).

### 2.4 MSN Change Generalized to Other Levels of Brain Organization

The connected regions in MSN have been confirmed to be more likely to belong to the same cytoarchitectonic class,^[^ ^8^ ^]^ and brain structure has been determined to constrain function. Therefore, we assigned brain regions to von Economo atlas with seven cytoarchitectonic classes (Supporting Information S09)^[^ ^19^ ^]^ and Yeo atlas with seven brain functional networks (Supporting Information S10).^[^ ^20^ ^]^ For von Economo cytoarchitectonic classes, acute mTBI patients showed the increased MS in the agranular cortex and frontal cortex, and decreased MS in the cerebral polar cortex (all for *p* < 0.05, FDR corrected) (Supporting Information S09, Figure S8A). Aligning with modular solutions, the agranular cortex was mainly located in module 7 and the cerebral polar cortex was located in module 3. Chronic mTBI patients showed decreased MS in the granular cortex mainly located in module 6 (*p* < 0.0001, FDR corrected) (Supporting Information S09, Figure S8C). For Yeo functional networks, the most significant increased MS in acute mTBI was found in the default mode network (DMN) (*p* = 0.0002, FDR corrected), and decreased MS were observed in the visual (VIS) network (*p* < 0.0001, FDR corrected) (Supporting Information S10, Figure S9A). The VIS network was concentrated in the module 3, while the DMN network was widely distributed in module 1, 5, and 7. For the chronic mTBI, decreased MS were found in the sensorimotor network (SMN, *p* = 0.048, FDR corrected) (Supporting Information S10, Figure S9C). After dividing patients into three subgroups based on cognitive impairment, consistent with the results of modularity, altered MS changes in cytoarchitectonic classes or functional networks only presented in the patients with no or single domain of cognitive impairment (Supporting Information S09 and S10, Figures S8B,D and S9B).

### 2.5 MSN Abnormalities in Pediatric mTBI

**Figure 6:**
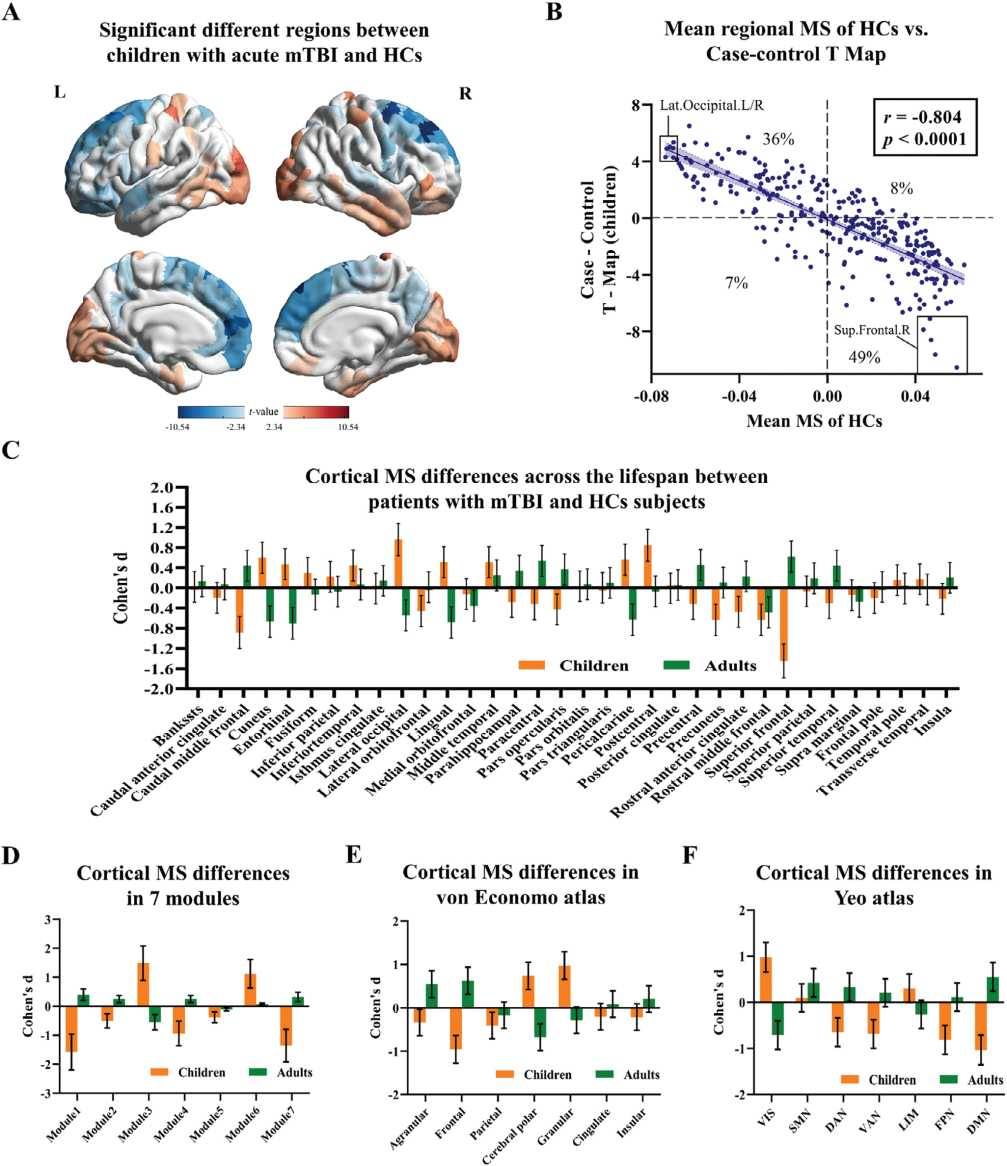
Morphological changes in pediatric mTBI. A) The regions with significant reginal MS alteration in children with mTBI compared with HCs children (for all *p* < 0.05, FDR corrected). B) The correlation between mean regional MS of HCs and case‐control T‐Map. Most regions exhibited decoupling (49%) and dedifferentiation (36%) in pediatric mTBI. C–F) show the cohen's d effect sizes with 95% confidence intervals for case‐control differences in cortical reginal MS classified by cortical regions, morphological mudules, von Economo cytoarchitectonic classes, and Yeo functional networks of children and adults. The greater differences of reginal MS in most regions were found in children compared with adults. MS, morphological similarity; VIS, visual; SMN, sensorimotor network; DAN, dorsal attention network; VAN, ventral attention network; LIM, limbic; FPN, frontoparietal network; DMN, default mode network.

Pediatric mTBI patients (N = 30) and matched controls (N = 31) were also included to reveal the morphological pattern in the developing brain after the mTBI exposure. The MSNs were extracted using the seven morphometric features derived from the T1w image. The global and regional MS of the pediatric mTBI were derived from MSN using the same method used in the adult mTBI. We also divided the global MS and regional MS of healthy children into the test group and replicated group to verify the consistency of MS (Supporting Information S11, Figure S10). Globally, pediatric mTBI presented decreased MS, compared with matched HCs (*p* = 0.0004) (Figure S11, Supporting Information). Regionally, the MS ranged from −0.07 to 0.06 in HCs and from −0.05 to 0.04 in pediatric mTBI (Figure S12, Supporting Information). Increased regional MS were mainly found in the lateral occipital lobe while decreased regional MS were mainly found in the superior frontal lobe (**Figure** **6**A, Supporting Information S12, Table S10). Correlation analysis between the mean MS of HCs and case‐control T‐Map was conducted to verify the different pattern of differentiation in the early developing brains compared with mature brains. Significant negative correlation (*r* = −0.804, *p* < 0.0001, Figure 6B) indicated the decoupling in 49% of regions and dedifferentiation in 36% of regions in the individuals with pediatric mTBI. The strong and significant case‐control difference of MS in most of the cytoarchitectonic classes and Yeo functional networks revealed the widespread cortical morphological changes in the pediatric mTBI (Supporting Information S13, Figure S13).

Effect size Cohen's d with 95% confidence interval were used to compare the case‐control differences in both children and adults. Since the pediatric cohort had no DTI datasets, we constructed the MSN using the seven features for both adult and pediatric TBI. Previous research has also proven the stability of the MSN after removing DTI features.^[^ ^21^ ^]^ Across the lifespan, case‐control differences of regional MS were more pronounced in pediatric mTBI than in adults. In pediatric mTBI compared with control children, the largest positive effect was found for lateral occipital cortex with a Cohen's d of 0.96 (*p* < 0.0001, FDR corrected), while the largest negative effect was found for superior frontal cortex with a Cohen's d of −1.45 (*p* < 0.0001, FDR corrected). The largest effect of adult mTBI were found for superior frontal cortex with a Cohen's d of 0.62 and for entorhinal cortex with a Cohen's d of −0.70 (*p* < 0.0001, FDR corrected) (Figure 6C). After dividing cortical areas into morphological mudules, cytoarchitectonic classes, and functional networks, broader and stronger changes in effect size were also found in pediatric mTBI patients compared with adult mTBI (Figure 6D–F).

### 2.6 Association Between Gene Expression and mTBI‐Related MSN Changes

**Figure 7:**
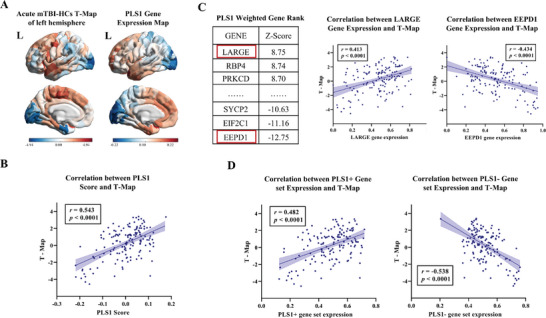
The association between gene expression and altered reginal MS in acute phase of mTBI. A) Case‐control T‐Map in the left hemisphere (left) and weighted gene expression map of PLS1 scores in the left hemisphere (right). B) The point plot shows the positive correlation between PLS1 gene expression map (PLS1 score) and case‐control T‐Map. C) The table shows the genes that contribute the most to the increase (i.e., LARGE) and decrease (i.e., EEPD1) of MS (left). The point plots show the positive correlation between LARGE gene (middle) and T‐Map as well as the negative correlation between EEPD1 gene and T‐Map (right). D) The point plot shows that the expression of the PLS1+ gene set is significantly positively correlated with the MS difference between mTBI patients and HCs (left), while the expression of the PLS1‐ gene set is significantly negatively correlated with the MS difference (right). PLS, Partial least squares.

We used AHBA (http://human.brain‐map.org) transcription profiles to generate a gene expression matrix for regions in the left hemisphere (152 regions × 10 027 genes). PLS regression^[^ ^22^ ^]^ was used to identify genes whose transcriptional profiles were significantly correlated with MS changes in the adult mTBI. The first component of the PLS (PLS1) represented the linear combination of transcriptional profiles mostly captured the changes of MS in the acute adult mTBI (*r* = 0.543, *p* < 0.0001) (**Figure** **7**A,B), which explained 16% of the variance (*p* < 0.0001). Based on the normalized PLS1 weighted Z score, we obtained 467 PLS+ genes (Z score > 4.56) and 1091 PLS‐genes (Z score < −4.56) (all for *p* < 0.05, Bonferroni corrected) (Figure 7C). PLS+ gene set contributed most to the increased MS, whose mean gene expression value was significantly positively correlated with T‐Map (*r* = 0.482, *p* < 0.0001). PLS‐ gene set contributed the most to the decreased MS, whose mean gene expression value was significantly negatively correlated with T‐Map (*r* = −0.538, *p* < 0.0001) (Figure 7D).

**Figure 8:**
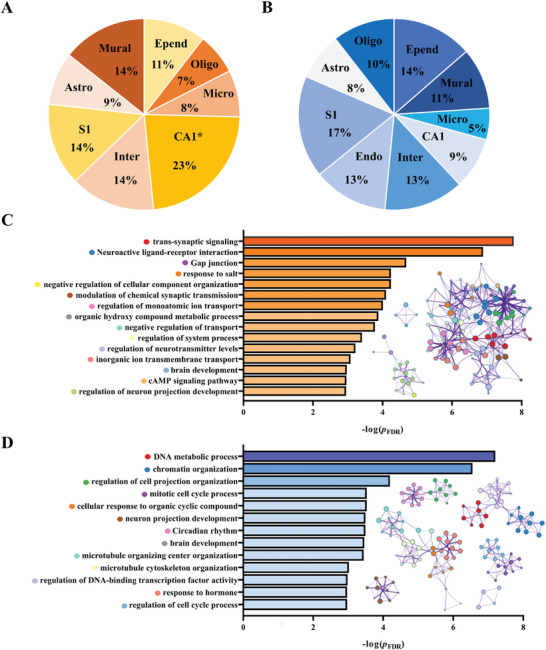
The enrichment of PLS1 gene sets. A) The proportion of the overlapping genes of PLS+ and cell‐type‐specific genes in each cell type. The PLS1+ genes showed significant specific expression in CA1 pyramidal cells. B) The proportion of the overlapping genes of PLS‐ and cell‐type‐specific genes in each cell type. PLS‐ genes did not express significantly in any cell type. C,D) show the bar and the network of the significant GO biological processes and KEGG pathways enrichment of PLS+ and PLS‐. The bars are colored by the ‐log(*p*_FDR_) value, and the networks are colored by cluster‐ID. All the GO and KEGG enrichment analyses were implemented using the Metascape online tool (https://metascape.org).

We identified 77 up‐regulated differentially expressed genes (DEGs) and 63 down‐regulated DEGs between TBI and HCs using the GSE2871 gene expression microarray (Supporting Information S14, Figure S14). Enrichment analysis showed the significant overlap between PLS+ genes and down‐regulated DEGs (*p* = 0.034, Bonferroni corrected, Supporting Information S15, Table S11), indicated that the gene set significantly correlated with increased MS was overexpressed in TBI‐related down‐regulated genes. Furthermore, we calculated the overlapped genes between PLS1 gene sets and nine cell‐type‐specific gene sets (Supporting Information S16). Only PLS+ gene represented significant specific expression in the CA1 pyramidal cells (*p* = 0.011, Bonferroni corrected) (**Figure** **8**A and Table S12, Supporting Information). The enrichment of PLS‐ genes was not observed in any type of cells Figure 8B and Table S13, Supporting Information).

Gene enrichment analyses were conducted to explore the biological processes involved in the PLS1 gene sets. GO biological process analysis and KEGG pathway analysis showed that PLS+ genes were mainly enriched in the neuron‐specific terms including trans‐synaptic signaling biological process, neuroactive ligand‐receptor interaction biological process, and neuroactive ligand‐receptor interaction pathway (Figure 8C), while the PLS‐ genes were mainly enriched in DNA‐specific terms including DNA metabolic process and chromatin organization process (Figure 8D). Notably, MSN‐related abnormal gene expression only occurred in the acute adult mTBI. The transcriptional profiling is incapable of significantly explaining the altered MS in chronic adult mTBI, which may indicate the pathophysiological recovery in the chronic phase of mTBI (Supporting Information S17, Figure S15). Our findings revealed the connections between MSN change patterns and specific cell types, as well as the biological processes of related genes.

## 3 Discussion

The present study innovatively introduces the MSN, serving as an intermediary to construct a framework that integrates clinical performances, neuroimaging phenotypes, and gene expression in mTBI. The MSN modular parcellation pattern, derived from a large sample of healthy individuals, effectively represents subject‐specific morphometric patterns. Validation of abnormal MSN patterns in both adult and pediatric mTBI cases highlights the diverse TBI‐related morphological changes throughout the lifespan. In particularly, the polarizing separation of regional MS across modules was associated with better cognitive performance post‐injury. The combined analysis of mTBI‐related cortical MS abnormalities and gene expression showed that the cortical MS remodeling is associated with the expression of genes. These genes highly expressed in CA1 pyramidal cells and involved in neuron‐specific biological process including trans‐synaptic signaling, suggesting the possible biological driver to TBI‐related cortical remodeling. These results established a link between macro‐ and micro‐scale phenotype through morphological changes, offering insights into the pathophysiological mechanisms and potential therapeutic targets in mTBI.

In contrast to using the single anatomical and morphometric features such as the cortical thickness, gray matter volume, and surface area, MSNs analysis can combine information across multiple cortical features in a single subject.^[^ ^23^ ^]^ Compared with the four MSN modules defined in the adolescents,^[^ ^8^ ^]^ the orbitofrontal cortex and the insula was identified as an independent component in the adult population. Unlike the high MS observed in most parts of the frontal lobe, the orbitofrontal cortex exhibited low MS, likely due to its distinctive histological properties, including numerous subregions characterized by varying cellular anatomy of cortical layers.^[^ ^24^ ^]^ Additionally, the unique slower growth pattern and divergent neuronal migration trajectory of the insula may lead to the gradually separated morphology.^[^ ^25^ ^]^

The occipital module with low MS in controls decreased their MS in mTBI patients (Hyperdifferentiation), which is probably indicative of reduced anatomical connectivity to and from the more differentiated cortical regions. The frontal modules with high MS in healthy controls increased their MS in patients with mTBI (Hypercoupling), which suggests a high degree of correspondence between them regarding cytoarchitectonic and myeloarchitectonic features. In line with the “hyperconnectivity hypothesis”, TBI is confirmed to associate with increased connectivity highly presented in transmodel regions that support cognition.^[^ ^26^ ^]^ The hyperconnectivity or hypercoupling is likely to be metabolically costly.^[^ ^27^ ^]^ Similarly, the increased MS in the frontal module of mTBI may suggest more tightly anatomical connections patterns in response to a damaged neurobiological substrate. Neurobiological damage confers less adaptability in regulating activity levels among network nodes. This may result in a simplified morphological architecture characterized by increased morphological coherence between network areas as observed across studies. Intriguingly, the increased MS in the frontal modules and the decreased MS in the occipital module were observed only in patients with no cognitive impairment or minimal cognitive impairment. Consistently, previous connectome‐based recovery mechanism indicated that the adaptive increase of structural connectome segregation was related to better cognitive recovery after mTBI.^[^ ^28^ ^]^ Convergent neurobiological evidence has suggested that the cortical organization commonly spans from primary and unimodal somatosensory cortices, to multimodal cortices, and finally to transmodal high‐order functional cortices, forming the hierarchical cortical axis of organization.^[^ ^29^ ^]^ The occipital module, dominated by primary and unimodal cortices, and the frontal module, characterized by high‐order cortices, are located at opposite ends of the cortical axis. Their segregation may imply a cortical reorganization mechanism after injury, but still need further researches.

The MS change patterns in acute mTBI were not permanent but reversed in the chronic phase, characterized by decreased MS in typically high MS regions and increased MS in highly differentiated regions. The decoupling and dedifferentiation patterns in the MS reflected the sustained damages to brain morphology during the chronic phase. The high‐order functional regions are more likely to be disturbed and change their connectivity in different forms at different stages of TBI.^[^ ^26^, ^30^ ^]^ Therefore, the decreases in MS during chronic mTBI may reflect the pattern of decreases in axonal connectivity of high‐order regions with the rest of the cortex.^[^ ^31^ ^]^ Although the presence of diverse regions exhibiting altered MS in the chronic phase, the MSN exhibits comprehensive recovery at the system level, indicating the maintain of system‐level normality through morphological reconfiguration in mTBI.^[^ ^32^ ^]^ Additionally, examining the brain MS changes in pediatric TBI allowed us to understand the ongoing maturational changes of substantial plasticity and windows of vulnerabilities. The loss of whole brain volume, structural integrity, and cellular integrity have been reported in the pediatric TBI.^[^ ^33^ ^]^ Compared with adult mTBI, pediatric mTBI exhibited more extensive and significant regional MS changes in both the frontal and occipital cortex. However, large‐sample and longitudinal studies are still needed to explore the pathophysiological mechanism underlying the abnormal MS patterns in pediatric TBI.

Recent efforts to understand the impact of genes on diseases and to study the relationship among neuroimaging features, genome, and molecular features have focus on garnering new insights from merging different data types using image genomics.^[^ ^34^ ^]^ To connect the MS changes in mTBI to the gene expression, cellular substrate, and biological pathways, we further identify the weighted combination of genes that contributed the most to the case–control MS differences in patients with mTBI. We found that the 467 highly expressed genes in regions with increased MS (PLS+ gene set) were significantly overlapped with TBI‐related down‐regulated DEGs, highly expressed in CA1 pyramidal cells and involved in neuron‐specific biological processes. CA1 pyramidal cells located in hippocampal area CA1, as an important part of the synaptic transmission pathway, playing a necessary role in cognitive performances.^[^ ^35^ ^]^ Acute TBI has reported to increase the intrinsic excitability in the CA1 pyramidal neurons, neuronal loss, and synaptic dysfunction, further leading to cognitive impairments.^[^ ^36^ ^]^ CA1 pyramidal cells can receive positional information directly from the entorhinal cortex, forming the entorhinal‐CA1 memory circuit.^[^ ^37^ ^]^ Synaptic dysfunction of entorhinal‐CA1 pathway leads to a novel mechanism of cognitive impairment in AD mice.^[^ ^38^ ^]^ The entorhinal cortex is located in medial temporal lobe and commonly reported in the early stage of AD.^[^ ^39^ ^]^ MTBI is considered as an important risk factor for AD, and leads to significantly reduced cortical thickness in the entorhinal cortex.^[^ ^40^ ^]^ Along the same line, we found that the decreased MS of the entorhinal cortex was associated with better performance in multiple cognitive domains. The cross‐scale association analysis further constructs the “gene overexpression – MS abnormalities – clinical cognitive impairment” pathway anchored to CA1 pyramidal cells and the entorhinal cortex in mTBI. The overexcited CA1 pyramidal cells may lead to synaptic disorders, cause the abnormal MS and cognitive impairments. This cross‐scale pathway may provide insights into the development of novel therapeutic strategies for TBI patients. For instance, greater attention should be focused on the entorhinal cortex and CA1 pyramidal cells. Specifically, deep brain stimulation or deep transcranial magnetic stimulation may be used to preferentially stimulate neurons along the disrupted pathways in the entorhinal cortex. Additionally, newly designed drugs that target the inhibition of CA1 neuron excitability could be considered.

Notwithstanding its implication, there were several limitations in this study. First, for both the adult and pediatric mTBI dataset, we used healthy individuals as control group. Including a control group of orthopedic trauma patients in future work may help to understand the specific contribution of MSN to brain injury. Second, due to the limitations of the scan protocol, the pediatric mTBI study only used seven morphological features from T1w imaging to construct MSN. Although the consistency of MS in the control group has been confirmed, the inconsistent scan protocol may still affect the interpretation of results. Multimodal MRI sequences, clinical neuropsychological assessments, and long‐term follow‐up are required in future to improve the sensitivity and reliability of the analysis. Third, transcription data from the left hemisphere of six post‐mortem brains may not accurately establish the relationship between whole‐brain gene expression and MSN. In‐depth cross‐scale methods to establish more precise mapping relationships between gene transcription and neuroimaging phenotype is warranted.

In summary, we have constructed a cross‐scale framework utilizing MSN that bridges the gap between heterogeneous clinical performances and gene transcription in mTBI. Alterations in the gene expression, particularly those enriched in CA1 pyramidal cells, may lead to morphological phenotypic changes, ultimately leading to multidimensional cognitive impairments. These insights offer a comprehensive cross‐scale perspective on the pathophysiological mechanism of mTBI and may present novel treatment targets for patients affected by this condition.

## 4 Experimental Section

### Subjects

All participants provided written informed consent before the experimental procedures, and research protocols were approved by Institutional Review Boards at all sites and performed according to the Helsinki Declaration. The experimental design is shown in Figure S16 (Supporting Information).

### Subjects—HCP Dataset

The publicly available 3T MRI data provided by the HCP S900 release (http://www.humanconnectome.org) were used, which contains 897 young healthy adult subjects with available structural scans. Only 840 subjects (372 males, 468 females, all aged 22– 35 except for 6 subjects older than 36) with complete T1w (0.7 mm isotropic) and MRI‐weighted imaging (DWI) data (1.25 mm isotropic, TR = 5520 ms, TE = 89.5 ms, 90 diffusion gradient orientations, and b = 1000/2000/3000 s mm^−2^) uploaded were finally selected in the study.

### Subjects—Adult mTBI Dataset

The adult mTBI dataset included 112 consecutive patients (59 males, mean age 37.35 ± 13.91 years) with mTBI enrolled from the local emergency department and 66 sex‐, age‐, and education‐matched healthy control participants (31 males, mean age 39.26 ± 13.57 years) recruited through advertisements. At the initial visit, 78 patients (69.6%) were willing to participate in the follow‐up examinations, and finally, 47 (60.3%) of them completed the examinations at 6–12 months post‐injury. The inclusion and exclusion criteria of the dataset are shown in Supporting Information S18.

High‐resolution T1w and DTI scans were performed in a 3.0 T MRI scanner (GE 750) with a 32‐channel head coil at both initial patients (within 7 days post‐injury) and 6–12 months follow‐up patients, as well as HCs. T1w images were obtained for each subject with brain volume imaging (BRAVO) sequence (TR = 7.68 ms, TE = 3.428 ms, flip angle = 9°, FOV = 256 × 256 mm^2^, voxel size = 1 × 1 × 1 mm^3^, and slices = 188). A single‐shot spin echo‐based planar imaging sequence was used to obtain the DTI (TR = 8000 ms, TE = 68 ms, FOV = 256 × 256 mm^2^, matrix size = 128 × 128, voxel size = 2 × 2 × 2 mm^3^, and slices = 75). Thirty diffusion gradient orientations were acquired in DTI scans (b = 1000 s mm^−2^), with the b = 0 repeated 5 times.

The neurocognitive assessments were carried out within 48 h of MRI scans, included: i) Trail‐Making Test Part A (TMT‐A) and Wechsler Adult Intelligence Scale (WAIS)‐III Digital Symbol Coding score (DSC) to evaluate cognitive information processing speed;^[^ ^41^ ^]^ ii) Forward Digit Span (FDS) from the WAIS‐III measured memory span to assess executive functions;^[^ ^42^ ^]^ iii) Backward Digit Span (BDS) from the WAIS‐III measured memory span to represent working memory,^[^ ^42^ ^]^ and iv) Verbal Fluency Test (VFT) to examine executive function and language ability.^[^ ^43^ ^]^

To distinguish the degree of cognitive impairments, patients were classified based on the neuropsychological assessments into no‐domain impairment (no impairment, NI), single‐domain impairment (moderate impairment, MI), and multi‐domain impairments (severe impairment, SI) using the same approach as the previous study.^[^ ^18^ ^]^

### Subjects—Pediatric mTBI Dataset

The pediatric mTBI dataset included 30 patients (16 males, mean age 8.60 ± 2.62 years) with mTBI and 31 demography‐matched healthy control participants (12 males, mean age 9.19 ± 1.89 years). All children were aged 4–14 years. Except for the age range, the inclusion and exclusion criteria for mTBI in children were the same as those for mTBI in adults. T1w scans were performed in GE750 and PHILIPS Ingenia 3.0 T MRI scanner for all participants. For patients, T1w images were obtained with BRAVO sequence using GE750 scanner (TR = 8.488 ms, TE = 3.248 ms, flip angle = 9°, FOV = 256 × 256 mm^2^, and voxel size = 1 × 1 × 1 mm^3^). For a portion of HCs, T1w images were obtained with BRAVO sequence using GE750 scanner (TR = 7.68 ms, TE = 3.428 ms, flip angle = 9°, FOV = 256 × 256 mm^2^, and voxel size = 1 × 1 × 1 mm^3^). T1w images for another portion of HCs were obtained with 3D structural T1w Fast Field Echo (sT1W.3D.TFE) sequence using PHILIPS Ingenia scanner (TR = 8.27 ms, TE = 3.794 ms, flip angle = 8°, FOV = 256 × 256 mm^2^, and voxel size = 1 × 1 × 1 mm^3^).

### Pediatric mTBI Dataset—MRI Data Processing

All the MRI data had passed quality control before subsequent data processing. The HCP dataset was downloaded in a preprocessed format, including the output of HCP Freesurfer pipeline and minimally processed DWI.^[^ ^44^ ^]^ The following preprocessing steps were applied to data in the adult mTBI, pediatric mTBI and control samples:

### Pediatric mTBI Dataset—Structural MRI Data Preprocessing

Surface‐based morphometry analysis of T1w anatomical data was implemented using recon‐all command^[^ ^45^ ^]^ from FreeSurfer v7.2.0 (http://surfer.nmr.mgh.harvard.edu/) to reconstruct cortical surfaces and segment volume regions of interest (ROIs). Briefly, this process entailed basic image correction and transform, generating of labeled structural parcellation, establishing of spherical cerebral surface, spherical mapping, and cortical parcellation. The cortical parcellation statistical table for each individual was obtained based on Desikan–Killiany cortical parcellation with 68 cortical ROIs,^[^ ^17b^ ^]^ including the statistics of gray matter volume (GMV), surface area (SA), cortical thickness (CT), mean curvature (MC), gaussian curvature (GC), folding index (FI), and curvature index (CI) (Supporting Information S19, Table S14).

### Pediatric mTBI Dataset—Diffusion MRI Data Preprocessing

DTI data of the adult mTBI dataset were preprocessed using the FSL6.0.5 software package (http://www.fmrib.ox.ac.uk/fsl/index.html) following the standard procedure.^[^ ^46^ ^]^ Briefly, the preprocessing comprised the subsequent steps: i) removing skull and non‐brain tissues by using the BET tool in FSL; ii) removing head motion by aligning the 30 diffusion‐weighted volumes (b = 1000 s mm^−2^) to the non‐weighted scan (b = 0 s mm^−2^); iii) correcting the eddy‐current distortion on diffusion‐weighted volumes, and align the results with the non‐weighted image using affine transformation; iv) estimating the native fractional anisotropy (FA) and mean diffusivity (MD) image by fitting the diffusion tensor model at each voxel.

### Pediatric mTBI Dataset—Morphological Feature Extraction

The Desikan–Killiany atlas^[^ ^17b^ ^]^ was split from 68 regions into 308 spatially contiguous and almost equal‐sized (≈5 cm) ROIs using the backtracking algorithm according to the previous study.^[^ ^17a^ ^]^ The backtracking algorithm included three steps: i) selecting a random seed vertex located on the periphery of the Desikan–Killiany atlas; ii) adding the nearest vertices to the seed within the Desikan–Killiany atlas until the size of the new parcel reached 5 cm^2^; and iii) excluding the new parcel and repeating from the first step using a different seed if any remaining vertices in the Desikan–Killiany atlas become isolated. The resulting atlas with 308 parcels (DK‐308 atlas) maintained a higher cortical resolution while ensuring uniform size for each parcel. The DK‐308 atlas was transformed from the surface of the Freesurfer standard template (fsaverage space) to native surface. The seven features (GMV, SA, CT, MC, GC, FI, and CI) from T1w images were then extracted from individual DK‐308 atlas, as gray matter metrics and curvature metrics. The FA and MD maps obtained through diffusion MRI preprocessing were mapped from diffusion MRI volume to native surface. The FA and MD statistics for DK‐308 atlas were then obtained as myelination metrics.

### Pediatric mTBI Dataset—Construction of MSN

Based on the nine features described above, each subject in HCP dataset and adult mTBI dataset obtained a 308 × 9 morphological feature matrix, while each subject in pediatric mTBI dataset obtained a 308 × 7 morphological feature matrix without FA and MD features. The morphological feature matrices were then normalized to unify the magnitude of different features using z‐score. The Pearson correlation of the normalized morphological feature matrix between each pair of cortical regions was performed to construct a 308 × 308 MSN for each subject.^[^ ^8^ ^]^ The global MS was gained by calculating the mean value of MSN. The regional MS was obtained by measuring the mean value of each row/column of MSN except self‐connection to represent the mean similarity between the selected region and all other ROIs.^[^ ^10a^ ^]^

### Generating Group Pattern of Modular Parcellation

The HCP dataset was used to generate the group‐level pattern of modularity based on MSN. In detail, the group average binarized MSN at 10% connection density was created for clustering. Louvain community modularity algorithm was then used to generate a stable large‐sample MSN community structure. According to the algorithm, each node in the MSN was initially assigned to an independent community. After moving each node to the community where its adjacent nodes were located, the modularity gain Δ*Q* was calculated using the following formula:

(1) $$\left\{\begin{array}{c} \Delta Q=\frac{1}{m}\left(A_{\mathrm{ij}}-\gamma \frac{k_{i}k_{j}}{m}\right),j\in N,j\neq i\\ \Delta Q=0,j=i \end{array}\right.$$

where *N* represents the number of nodes in the network, which was 308 in MSN; *m* is the number of edges in MSN; *A_ij_* is the edge weights between node *i* and its adjacent nodes *j*, which were set to 1 in the binarized MSN; γ is the adjustable resolution parameter for maintaining a suitable community size, which was set to 1.1 here; *k_i_* is the degree of node *i*; and *k_j_* is the degree of adjacent node *j*. High positive Δ*Q* implies that relocating the node to another community will substantially enhance the modularity of the network. Thus, when the maximum Δ*Q* > 0, node *i* is placed into the community where the adjacent node *j* with the largest Δ*Q* is located. The relocated node exhibits stronger connections with other nodes within the target community, while its connections with the original community were comparatively weaker. This relocation process continues until the movement of each node no longer increases Δ*Q*. Throughout the iterative process, the configuration of the γ parameter may impact the outcomes. The altered γ may lead to different numbers of resulting modules. As the γ changes, the more‐modules solution was the subdivision of the less‐modules solution (Supporting Information S03, Figure S1). The 308 × 308 group mean MSN was finally divided into seven spatially continuous modules as the group‐level morphological modularity pattern^[^ ^8^, ^47^ ^]^ (Supporting Information S20, Table S15).

### The Mapping of Transcription‐Imaging Association

The AHBA (http://human.brain‐map.org)^[^ ^48^ ^]^ transcriptional profiles consisting of expression measures of 20 737 genes from 6 postmortem subjects (5 male, mean age 42.50 ± 13.38 years) were used to bridge the gap between MSN and gene transcription in mTBI. Only considered left hemisphere in the analysis because not all donors of AHBA had samples in the right hemisphere. The preprocessing of AHBA data included six steps according to a previously published study:^[^ ^49^ ^]^ i) verifying probe‐to‐gene annotations; ii) filtering probes; iii) selecting representative probes; iv) mapping AHBA tissue samples to DKT‐308 atlas; v) normalizing expression measures; and vi) filtering gene‐set. Thus, the transcription matrix of the 10 027 probes on 152 left hemisphere regions (152 regions × 10027 genes) was obtained.

PLS regression^[^ ^22^ ^]^ was used to identify genes whose transcriptional profiles (152 × 10027 matrix as predictor variables) were significantly correlated with regional MS differences (152 [regions] × 1 [t‐value] matrix as response variables).^[^ ^10b^ ^]^ The first component of the PLS (PLS1) was the linear combination of transcriptional profiles most closely related to the regional MS difference in mTBI. Permutation test (1000 times) was then used to test the statistical significance of the variance explained by PLS1. For each gene, bootstrapping (1000 bootstrap samples) was used to correct the PLS1 weight as follows:

(2) $$PLS1W_{corr}=Z_{score}\left(\frac{PLS1W}{PLS1W_{BSE}}\right)$$

where *PLS*1*W_corr_* is the z‐score normalized corrected PLS1 weight, *PLS*1*W* is the original PLS1 weight, and *PLS*1*W_BSE_* is the bootstrap standard error of *PLS*1*W*. Genes with *PLS*1*W_corr_* (Z score) > 4.56 and *PLS*1*W_corr_* (Z score) < −4.56 (*p* < 0.05, Bonferroni corrected) were defined as PLS+ gene set and PLS‐ gene set, which represented the gene lists with the maximum contribution to the PLS1.^[^ ^10c^ ^]^

### Gene Enrichment Analysis—DEGs Enrichment Analysis

The gene expression microarray with serial number GSE2871 from GEO database (http://www.ncbi.nlm.nih.gov/geo) was used to explore TBI‐related DEGs. The dataset comprises 47 parietal cortex and hippocampus samples of rats, including 16 sham surgery samples without injury, 15 samples at 4‐h post‐TBI, and 16 samples at 24‐h post‐TBI. The LIMMA package of R software was used to analyze the up‐regulated and down‐regulated DEGs between TBI rats of 24 h post‐injury and the sham rats based on the principle of | log_2_ (fold change) | ≥ 1 and adjusted *p*‐value < 0.05. The DEGs were then mapped to human genes using the R package developed by Ogan Mancarci (https://github.com/oganm/homologene). The overlap between the PLS1 gene sets with up‐regulated and down‐regulated genes were measured to explore the enrichment of the PLS1 gene sets in DEGs.

### Gene Enrichment Analysis—Cell‐Type‐Specific Gene Enrichment Analysis

The lists of mouse genes expressed in nine specific cell types including ependymal, oligodendrocytes, microglia, CA1 pyramidal neurons, interneurons, endothelial, S1 pyramidal neurons, astrocytes, and mural^[^ ^50^ ^]^ were mapped to human genes as the cell‐type‐specific gene sets. The overlap between PLS1 gene sets and each cell‐type‐specific gene set was then calculated to identify the enrichment of PLS1 gene sets in each cell type.

### Gene Enrichment Analysis—Online Enrichment Analysis

Metascape online tool (https://metascape.org) was used to perform the automated enrichment analysis including GO biological processes enrichment and KEGG pathways enrichment for the PLS1 gene sets.

### Interpretation of Statistical Measures

The Shapiro–Wilk W‐test was used to test for normality distribution of all continuous variables. Chi‐square analyses were used to assess categorical variables. Age, sex, and years of education were regressed as covariates using linear model. Two‐sample *t*‐test and Mann–Whitney test were used to perform between‐group comparison of MSN. Analysis of variance (ANOVA) was run to compare the MSN difference among NI, MI, SI patients, and HCs. Partial correlation analysis was used to measure the correlated neuropsychological variables with the regional mean MS. Besides, one‐sample z‐tests were used to determine the threshold of PLS1 sets. Hypergometric test was used to evaluate the significance of DEGs enrichment and cell‐type‐specific gene enrichment. *p*‐value <0.05 was considered statistically significant, and FDR (false discovery rate) correction or Bonferroni correction was performed for multiple comparisons in all involved analyses.

## Conflict of Interest

The authors declare no conflict of interest.

## Supporting information



Supporting Information

## Data Availability

The data that support the findings of this study are available from the corresponding author upon reasonable request.
