# Supplementary material for: Cortical Morphometric Similarity Remodeling in Traumatic Brain Injury Links Cognitive Impairments with Transcriptional Changes and Type‐Specific Cells
Source: Adv Sci (Weinh). 2025 Feb 7;12(13):2415262. doi: 10.1002/advs.202415262 (PMC11967866; doi:10.1002/advs.202415262)
Supplement: Supplementary file 1 — Supporting Information [file ADVS-12-2415262-s001.docx]

Supporting Information

Cortical Morphometric Similarity Remodeling in Traumatic Brain Injury Links Cognitive Impairments with Transcriptional Changes and Type-specific Cells

Yizhen Pan, Zhuonan Wang, Xiang Zhang, Wenpu Zhao, Haonan Zhang, Xuan Li, Xiaoyan Jia, Qiuyu Ji, Bo Yin, Guanghui Bai, Tingting Wu, Zhiqi Lee, Jierui Ding, Lei Shi, Jie Zhang*, David H. Salat, Lijun Bai*

**S01: Demographic and cognitive characteristics of participants**

In the adult mTBI dataset, acute mTBI patients did not differ from HCs regarding to sex (*p* = 0.462), age (*p* = 0.373) and years of education (*p* = 0.092). Concerning neurocognitive tests, patients with mTBI presented significantly worse performances on Trail-Making Test Part A (TMT-A, *p* = 0.004), Digital Symbol Coding score (DSC, *p* = 0.005) and Verbal Fluency Test (VFT, *p* = 0.048). The details were summarized in **Table S1**.

**Table S1.** Demographic and cognitive characteristics for mild traumatic brain injury and healthy control participants in adults

| **Items** | **mTBI Group (n=112)** | **HCs Group (n=66)** | ***p* value** |
| --- | --- | --- | --- |
| Male, n (%) | 59 (52.7) | 31 (47.0) | 0.462 |
| Age, y | 37.35 ± 13.91 | 39.26 ± 13.57 | 0.373 |
| Education, y | 8.32 ± 4.11 | 9.71 ± 5.84 | 0.092 |
| TMT-A | 71.21 ± 54.58 | 51.83 ± 33.08 | 0.004 |
| DSC | 33.53 ± 16.70 | 41.09 ± 17.85 | 0.005 |
| FDS | 7.69 ± 1.51 | 8.02 ± 1.73 | 0.187 |
| BDS | 3.94 ± 1.54 | 4.38 ± 1.80 | 0.086 |
| VFT | 16.04 ± 5.67 | 17.83 ± 5.84 | 0.048 |

mTBI, mild traumatic brain injury; HCs, healthy controls; TMT-A, Trail making A; DSC, Digit Symbol Coding Score; FDS, Forward Digit Span; BDS, Backward Digit Span; VFT, Verbal Fluency Test.

In the pediatric mTBI dataset, there were also no significant differences between mTBI and HCs in basic demographic characteristics including sex (*p* = 0.252) and age (*p* = 0.316) (**Table S2**).

**Table S2.** Demographic Data for mild traumatic brain injury and healthy control participants in children

| **Items** | **mTBI Group (n=30)** | **HCs Group (n=31)** | ***p* value** |
| --- | --- | --- | --- |
| Male, n (%) | 16 (53.3) | 12 (38.7) | *0.252* |
| Age, y | 8.60 ± 2.62 | 9.19± 1.89 |  |

mTBI, mild traumatic brain injury; HCs, healthy controls.

**S02: Verify the stability of modular parcellation**

To verify the stability of the module parcellation obtained by Louvain community modularity algorithm, we evaluated altogether four network sparsity thresholds (T = 5%, 10%, 15%, 20%) and three resolution parameter values (γ = 1.0, 1.1, 1.2). The modularity under different network sparsity and resolution parameters are shown in **Figure S1**. The change of network sparsity hardly affects the stability of the module parcellation. For example, when γ = 1.0, the solution under four network sparsity levels were highly consistent. However, the altered γ causes the different numbers of modules. As the γ changes, the more-modules solution is the subdivision of the less-modules solution. For example, in the 5-module solution, the anterior cingulate cortex and its connected prefrontal cortex were divided as one module, whereas in the 7-module solution, they were divided into two separate modules. In addition, excessive modules may lead to isolated module region. For example, in the 9-module solution, the left superior frontal cortex (part11) was divided into an independent module.

**
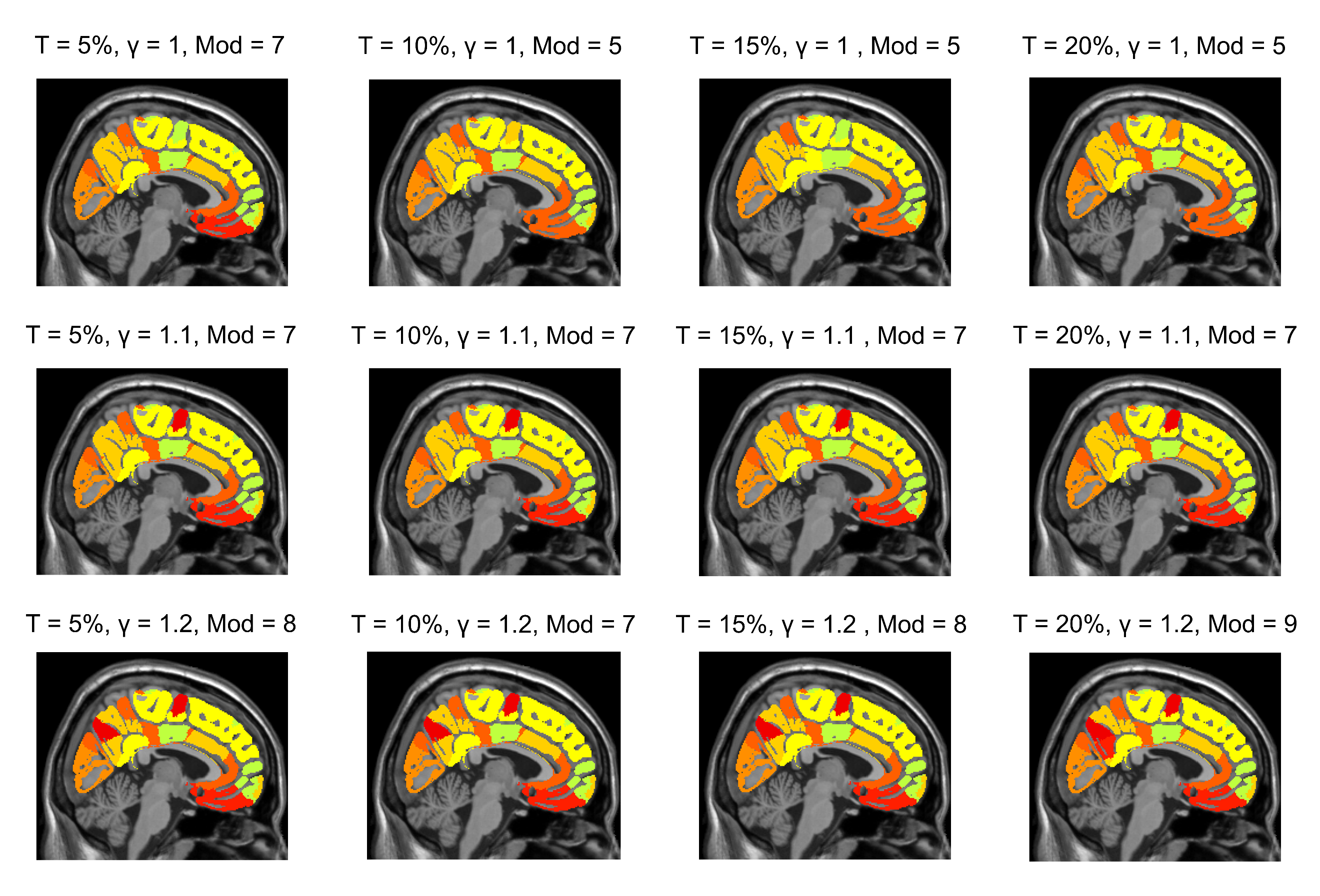
**

**Figure S1.** The modularity under four network sparsity thresholds (T) and three resolution parameter values (γ). Mod, the numbers of modules.

**S03: The regional MS in the 7 modules in HCP dataset**

To investigate the regional MS varies spatially across the brain, we calculated the distribution of regional MS in the 7 modules of HCP dataset. At the group level, regional MS varied greatly across different modules. Module 1 and module 7 areas had higher regional MS than the other modules, with values of 0.008 ± 0.006 and 0.006 ± 0.004, while module 2, module 3 and module 4 areas generally had weaker regional MS than the other modules, with values of -0.010 ± 0.012, -0.007 ± 0.004 and -0.027 ± 0.009 (**Figure S2**). These results indicated the higher MS in extensive frontal lobe regions and the lower MS in the insula, the occipital lobe and the orbitofrontal cortex.

**
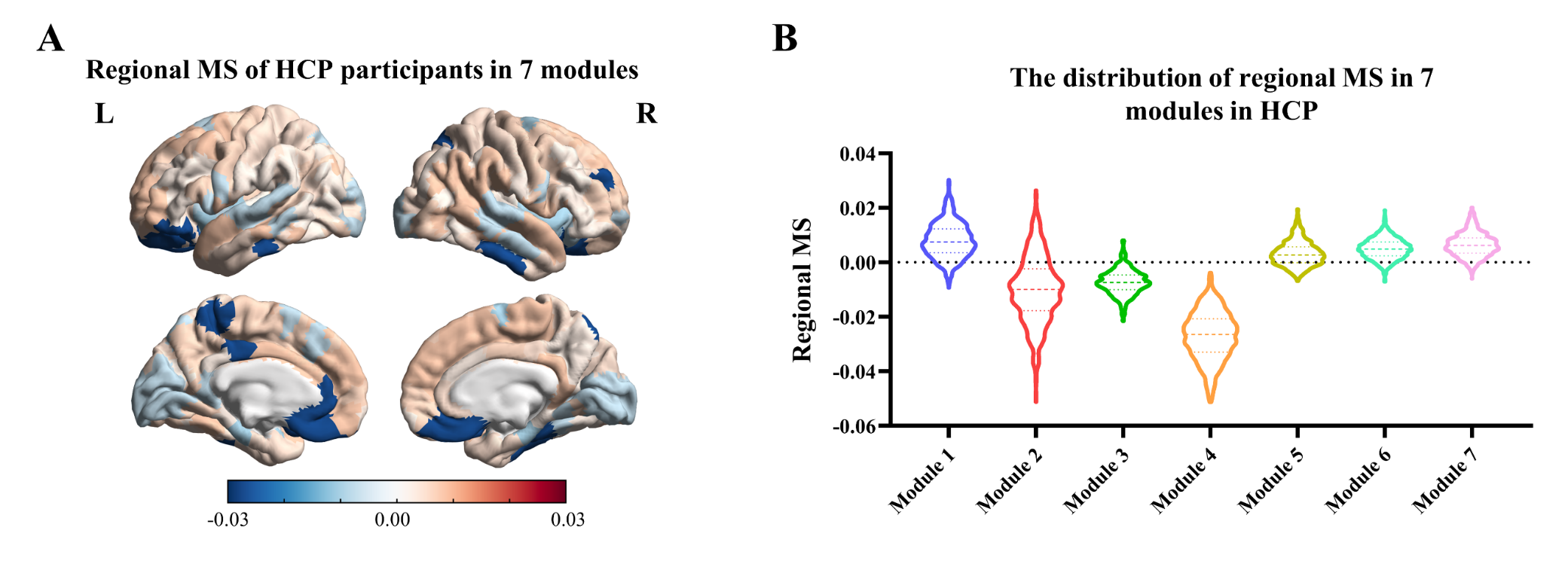
**

**Figure S2.** Regional MS of HCP participants in 7 modules.

**S04: Regions with significant differences in MS between adult mTBI and HCs**

Forty-five brain regions with altered regional MS were found in acute mTBI, including 22 increased regions and 23 decreased regions (all *p* < 0.05, FDR corrected). Among them, 16 regions (73%) with increased regional MS were located in module 1 and 7, while 17 regions (74%) with decreased regional MS were located in module 3. The positive t-value represented the increased regional MS in acute patients and the negative t-value represented the decreased regional MS. The specific information on abnormal regions is listed in **Table S3-S4**.

**Table S3.** The regions with significant increased regional MS (all *p* < 0.05, FDR corrected) in acute mTBI patients compared with HCs.

| Module | Regions | T-value | *P* value  (FDR) | *P* value  (Bonferroni) |
| --- | --- | --- | --- | --- |
| 1 | rh_superiorfrontal_part13 | 3.530 | 0.0136 | 0.1636 |
| 1 | lh_superiortemporal_part2 | 3.347 | 0.0199 | 0.3081 |
| 1 | rh_superiorfrontal_part7 | 3.175 | 0.0247 | 0.5452 |
| 1 | lh_superiortemporal_part7 | 3.135 | 0.0258 | 0.6197 |
| 1 | rh_superiorfrontal_part8 | 3.048 | 0.0307 | 0.8811 |
| 1 | rh_superiortemporal_part4 | 3.025 | 0.0307 | 0.8797 |
| 1 | rh_superiorfrontal_part10 | 3.018 | 0.0307 | 0.8994 |
| 1 | rh_parstriangularis_part2 | 3.011 | 0.0307 | 0.9195 |
| 1 | lh_superiortemporal_part4 | 2.742 | 0.0472 | 1.0000 |
| 7 | lh_paracentral_part2 | 3.394 | 0.0199 | 0.2622 |
| 7 | lh_precentral_part9 | 3.264 | 0.0203 | 0.4069 |
| 7 | lh_parsopercularis_part3 | 3.039 | 0.0307 | 0.8420 |
| 7 | lh_superiorfrontal_part6 | 2.939 | 0.0349 | 1.0000 |
| 7 | rh_precentral_part7 | 2.897 | 0.0374 | 1.0000 |
| 7 | rh_caudalmiddlefrontal_part4 | 2.846 | 0.0413 | 1.0000 |
| 7 | lh_precentral_part8 | 2.737 | 0.0199 | 1.0000 |
| 3 | rh_superiortemporal_part6 | 3.094 | 0.0283 | 0.7084 |
| 3 | rh_bankssts_part1 | 2.869 | 0.0396 | 1.0000 |
| 3 | rh_middletemporal_part6 | 2.775 | 0.0448 | 1.0000 |
| 6 | lh_lateralorbitofrontal_part4 | 3.289 | 0.0203 | 0.3742 |
| 6 | rh_superiorparietal_part5 | 2.926 | 0.0352 | 1.0000 |
| 2 | rh_superiorfrontal_part3 | 2.779 | 0.0453 | 1.0000 |

lh, left hemisphere; rh, right hemisphere. T-values were obtained from regional MS data (regressed age, sex, and years of education) using two-sample t-tests. All *p* values were adjusted by FDR correction and Bonferroni correction, and were determined based on 2-sided tests.

**Table S4.** The regions with significant decreased regional MS (all *p* < 0.05, FDR corrected) in acute mTBI patients compared with HCs.

| Module | Regions | T-value | *P* value  (FDR) | *P* value  (Bonferroni) |
| --- | --- | --- | --- | --- |
| 3 | rh_lingual_part2 | -4.940 | 0.0006 | 0.0006 |
| 3 | rh_lingual_part4 | -4.273 | 0.0024 | 0.0097 |
| 3 | lh_lateraloccipital_part5 | -4.311 | 0.0024 | 0.0083 |
| 3 | rh_lateraloccipital_part7 | -3.950 | 0.0070 | 0.0348 |
| 3 | lh_lateraloccipital_part3 | -3.880 | 0.0076 | 0.0455 |
| 3 | lh_lingual_part2 | -3.720 | 0.0118 | 0.0825 |
| 3 | lh_lingual_part6 | -3.683 | 0.0118 | 0.0945 |
| 3 | lh_lingual_part4 | -3.641 | 0.0122 | 0.1099 |
| 3 | rh_lateraloccipital_part1 | -3.549 | 0.0136 | 0.1528 |
| 3 | lh_lateraloccipital_part9 | -3.319 | 0.0199 | 0.3383 |
| 3 | lh_lateraloccipital_part8 | -3.372 | 0.0199 | 0.2826 |
| 3 | rh_lingual_part6 | -3.162 | 0.0247 | 0.5691 |
| 3 | rh_lateraloccipital_part8 | -3.166 | 0.0247 | 0.5610 |
| 3 | rh_cuneus_part3 | -2.989 | 0.0317 | 0.9842 |
| 3 | lh_cuneus_part2 | -2.976 | 0.0320 | 1.0000 |
| 3 | rh_cuneus_part2 | -2.811 | 0.0435 | 1.0000 |
| 3 | lh_pericalcarine_part2 | -2.791 | 0.0448 | 1.0000 |
| 6 | lh_lateraloccipital_part7 | -4.568 | 0.0014 | 0.0028 |
| 6 | lh_entorhinal_part1 | -3.602 | 0.0126 | 0.1263 |
| 6 | rh_temporalpole_part1 | -3.333 | 0.0199 | 0.3228 |
| 6 | rh_entorhinal_part1 | -3.270 | 0.0203 | 0.3984 |
| 5 | lh_supramarginal_part6 | -2.783 | 0.0448 | 1.0000 |
| 7 | lh_lateraloccipital_part6 | -2.828 | 0.0424 | 1.0000 |

lh, left hemisphere; rh, right hemisphere. T-values were obtained from regional MS data (regressed age, sex, and years of education) using two-sample t-tests. All *p* values were adjusted by FDR correction and Bonferroni correction, and were determined based on 2-sided tests.

We then found 86 brain regions with altered regional MS in chronic mTBI, including 34 increased regions and 52 decreased regions. The specific information of abnormal brain regions was listed in **Table S5 and Table S6**.

**Table S5.** The regions with significant increased regional MS (all p < 0.05, FDR corrected) in chronic mTBI patients compared with HCs.

| Module | Regions | T-value | *P* value  (FDR) | *P* value  (Bonferroni) |
| --- | --- | --- | --- | --- |
| 6 | lh_temporalpole_part1 | 5.588 | 1.70E-05 | 5.11E-05 |
| 6 | lh_inferiortemporal_part6 | 3.422 | 0.0071 | 0.2680 |
| 6 | lh_lateraloccipital_part2 | 3.378 | 0.0078 | 0.3109 |
| 6 | rh_temporalpole_part1 | 3.203 | 0.0105 | 0.5470 |
| 6 | lh_inferiortemporal_part3 | 3.192 | 0.0105 | 0.5654 |
| 6 | rh_inferiortemporal_part4 | 3.160 | 0.0110 | 0.6271 |
| 6 | lh_superiorparietal_part7 | 3.056 | 0.0144 | 0.8653 |
| 6 | rh_rostralanteriorcingulate_part1 | 2.770 | 0.0290 | 1.0000 |
| 6 | lh_inferiortemporal_part1 | 2.673 | 0.0361 | 1.0000 |
| 6 | lh_fusiform_part4 | 2.660 | 0.0368 | 1.0000 |
| 5 | lh_inferiorparietal_part7 | 3.815 | 0.0033 | 0.0690 |
| 5 | rh_parsorbitalis_part1 | 3.633 | 0.0048 | 0.1313 |
| 5 | lh_lateraloccipital_part4 | 3.427 | 0.0071 | 0.2641 |
| 5 | rh_inferiorparietal_part9 | 3.186 | 0.0105 | 0.5783 |
| 5 | lh_inferiorparietal_part6 | 2.981 | 0.0175 | 1.0000 |
| 5 | lh_frontalpole_part1 | 2.913 | 0.0200 | 1.0000 |
| 5 | lh_middletemporal_part2 | 2.701 | 0.0344 | 1.0000 |
| 7 | rh_lateraloccipital_part9 | 3.618 | 0.0048 | 0.1383 |
| 7 | rh_lateraloccipital_part5 | 3.531 | 0.0059 | 0.1862 |
| 7 | rh_rostralmiddlefrontal_part1 | 3.330 | 0.0085 | 0.3634 |
| 7 | lh_lateraloccipital_part6 | 3.260 | 0.0095 | 0.4556 |
| 7 | lh_rostralmiddlefrontal_part4 | 3.160 | 0.0110 | 0.6260 |
| 7 | lh_inferiorparietal_part8 | 2.933 | 0.0190 | 1.0000 |
| 3 | lh_lateraloccipital_part9 | 3.268 | 0.0095 | 0.4448 |
| 3 | rh_rostralmiddlefrontal_part2 | 3.035 | 0.0151 | 0.9231 |
| 3 | rh_superiortemporal_part2 | 2.543 | 0.0465 | 1.0000 |
| 3 | rh_middletemporal_part1 | 2.518 | 0.0482 | 1.0000 |
| 4 | lh_rostralanteriorcingulate_part1 | 4.361 | 6.10E-04 | 0.0090 |
| 4 | rh_inferiortemporal_part5 | 3.273 | 0.0095 | 0.4370 |
| 4 | rh_inferiortemporal_part3 | 2.971 | 0.0175 | 1.0000 |
| 4 | lh_posteriorcingulate_part1 | 2.535 | 0.0468 | 1.0000 |
| 1 | lh_inferiortemporal_part4 | 3.403 | 0.0073 | 0.2855 |
| 1 | rh_supramarginal_part5 | 3.192 | 0.0105 | 0.5669 |
| 1 | rh_inferiorparietal_part8 | 2.975 | 0.0175 | 1.0000 |

lh, left hemisphere; rh, right hemisphere. T-values were obtained from regional MS data (regressed age, sex, and years of education) using two-sample t-tests. All *p* values were adjusted by FDR correction and Bonferroni correction, and were determined based on 2-sided tests.

**Table S6.** The regions with significant decreased regional MS (all p < 0.05, FDR corrected) in chronic mTBI patients compared with HCs.

| Module | Regions | T-value | P-value (FDR) | P-value (Bonf) |
| --- | --- | --- | --- | --- |
| 6 | lh_postcentral_part6 | -5.345 | 3.91E-05 | 0.0002 |
| 6 | rh_postcentral_part3 | -4.435 | 5.60E-04 | 0.0067 |
| 6 | lh_postcentral_part8 | -4.383 | 6.10E-04 | 0.0091 |
| 6 | rh_precuneus_part4 | -3.791 | 0.0034 | 0.0754 |
| 6 | lh_lateralorbitofrontal_part4 | -3.773 | 0.0035 | 0.0804 |
| 6 | lh_postcentral_part3 | -3.609 | 0.0048 | 0.1434 |
| 6 | rh_supramarginal_part1 | -3.525 | 0.0060 | 0.1901 |
| 6 | rh_superiorparietal_part1 | -3.420 | 0.0071 | 0.2701 |
| 6 | rh_lateraloccipital_part6 | -3.361 | 0.0080 | 0.3279 |
| 6 | lh_rostralmiddlefrontal_part7 | -3.350 | 0.0081 | 0.3408 |
| 6 | lh_postcentral_part5 | -3.291 | 0.0094 | 0.4151 |
| 6 | rh_precuneus_part3 | -3.285 | 0.0094 | 0.4209 |
| 6 | lh_supramarginal_part7 | -3.081 | 0.0138 | 0.8013 |
| 6 | rh_postcentral_part1 | -3.077 | 0.0138 | 0.8161 |
| 6 | lh_superiorparietal_part9 | -2.786 | 0.0280 | 1.0000 |
| 1 | rh_superiorfrontal_part10 | -4.571 | 3.56E-04 | 0.0039 |
| 1 | rh_parstriangularis_part2 | -4.390 | 6.10E-04 | 0.0080 |
| 1 | rh_postcentral_part2 | -4.200 | 9.82E-04 | 0.0167 |
| 1 | lh_superiortemporal_part7 | -4.000 | 0.0018 | 0.0353 |
| 1 | rh_paracentral_part3 | -3.634 | 0.0048 | 0.1308 |
| 1 | rh_precentral_part1 | -3.490 | 0.0063 | 0.2138 |
| 1 | rh_paracentral_part1 | -3.243 | 0.0098 | 0.4810 |
| 1 | lh_superiorfrontal_part11 | -2.699 | 0.0344 | 2.4742 |
| 1 | rh_superiorfrontal_part4 | -2.630 | 0.0395 | 3.0020 |
| 1 | rh_parsopercularis_part1 | -2.596 | 0.0417 | 3.2972 |
| 1 | rh_superiortemporal_part1 | -2.546 | 0.0465 | 3.7794 |
| 3 | lh_lingual_part5 | -5.619 | 1.70E-05 | 4.45E-05 |
| 3 | rh_pericalcarine_part1 | -4.939 | 1.23E-04 | 0.0009 |
| 3 | rh_lingual_part5 | -4.735 | 2.51E-04 | 0.0020 |
| 3 | rh_cuneus_part3 | -4.133 | 0.0011 | 0.0215 |
| 3 | rh_lingual_part1 | -3.628 | 0.0048 | 0.1332 |
| 3 | lh_pericalcarine_part2 | -3.499 | 0.0063 | 0.2072 |
| 3 | rh_superiortemporal_part6 | -3.482 | 0.0063 | 0.2192 |
| 3 | lh_insula_part4 | -2.612 | 0.0404 | 3.1536 |
| 3 | lh_superiortemporal_part6 | -2.575 | 0.0437 | 3.4946 |
| 3 | lh_cuneus_part1 | -2.508 | 0.0486 | 4.1815 |
| 5 | lh_precuneus_part3 | -4.169 | 0.0010 | 0.0188 |
| 5 | lh_supramarginal_part2 | -3.762 | 0.0035 | 0.0834 |
| 5 | rh_parahippocampal_part1 | -3.191 | 0.0105 | 0.5683 |
| 5 | lh_parahippocampal_part2 | -2.965 | 0.0176 | 1.1439 |
| 5 | lh_rostralmiddlefrontal_part1 | -2.619 | 0.0402 | 3.0958 |
| 5 | lh_parstriangularis_part2 | -2.516 | 0.0482 | 4.0926 |
| 7 | lh_lingual_part1 | -5.912 | 1.17E-05 | 1.17E-05 |
| 7 | lh_superiortemporal_part1 | -5.162 | 6.01E-05 | 0.0003 |
| 7 | lh_lingual_part3 | -3.680 | 0.0045 | 0.1114 |
| 7 | lh_precuneus_part7 | -2.852 | 0.0235 | 1.5953 |
| 7 | rh_lingual_part3 | -2.683 | 0.0355 | 2.5877 |
| 4 | rh_medialorbitofrontal_part2 | -5.169 | 0.0004 | 0.0004 |
| 4 | lh_medialorbitofrontal_part1 | -4.642 | 0.0030 | 0.0030 |
| 4 | lh_precuneus_part2 | -4.234 | 0.0146 | 0.0146 |
| 2 | rh_parahippocampal_part2 | -4.575 | 3.56E-04 | 0.0038 |
| 2 | rh_insula_part4 | -3.224 | 0.0102 | 0.5114 |

lh, left hemisphere; rh, right hemisphere. T-values were obtained from regional MS data (regressed age, sex, and years of education) using two-sample t-tests. *p* values were adjusted by FDR correction and Bonferroni correction, and were determined based on 2-sided tests. Bonf, Bonferroni.

**S05: Lock in the ROIs to explore the relation with cognitive assessments**

The DKT-308 atlas used in the current study split the Desikan-Killiany atlas from 68 regions into 308 spatially contiguous ROIs. The corresponding relationship between Desikan Killiany atlas and DKT-308 atlas is shown in **Table S7 and Table S8**. The regions in Desikan-Killiany atlas with more than 30% MS increased or decreased subregions were regarded as ROIs. Therefore, for acute mTBI, superior temporal was selected as the ROI with increased MS. Cuneus, entorhinal cortex, lateral occipital cortex, lingual gyrus and temporal pole were selected as the ROIs with decreased MS (**Table S7**).

**Table S7.** The proportion of altered regions in Desikan-Killiany atlas of acute mTBI.

| Regions in Desikan Killiany atlas | Label in DKT-308 atlas | Proportion of increased regions (%) | Proportion of decreased regions (%) |
| --- | --- | --- | --- |
| Bankssts | 1-2, 153-154 | 25 | 0 |
| Caudal anterior cingulate | 3, 155 | 0 | 0 |
| Caudal middle frontal | 4-7, 156-159 | 12.5 | 0 |
| Cuneus | 8-9, 160-162 | 0 | 60 |
| Entorhinal | 10, 163 | 0 | 100 |
| Fusiform | 11-15, 164-168 | 0 | 0 |
| Inferior parietal | 16-23, 169-178 | 0 | 0 |
| Inferior temporal | 24-29, 179-183 | 0 | 0 |
| Isthmus cingulate | 30-31, 184-185 | 0 | 0 |
| Lateral occipital | 32-40, 186-194 | 0 | 50 |
| Lateral orbitofrontal | 41-44, 195-198 | 12.5 | 0 |
| Lingual | 45-50, 199-204 | 0 | 50 |
| Medial orbitofrontal | 51-53, 205-207 | 0 | 0 |
| Middle temporal | 54-58, 208-213 | 9.1 | 0 |
| Parahippocampal | 59-60, 214-215 | 0 | 0 |
| Paracentral | 61-63, 216-218 | 16.7 | 0 |
| Pars opercularis | 64-66, 219-221 | 16.7 | 0 |
| Pars orbitalis | 67, 222 | 0 | 0 |
| Pars triangularis | 68-69, 223-225 | 20 | 0 |
| Pericalcarine | 70-71, 226-228 | 0 | 20 |
| Postcentral | 72-79, 229-236 | 0 | 0 |
| Posterior cingulate | 80-81, 237-238 | 0 | 0 |
| Precentral | 82-90, 239-247 | 16.7 | 0 |
| Precuneus | 91-97, 248-254 | 0 | 0 |
| Rostral anterior cingulate | 98, 255 | 0 | 0 |
| Rostral middle frontal | 99-108, 256-265 | 0 | 0 |
| Superior frontal | 109-121, 266-278 | 23.1 | 0 |
| Superior parietal | 122-131, 279-288 | 5 | 0 |
| Superior temporal | 132-138, 289-294 | 38.5 | 0 |
| Supra marginal | 139-145, 295-301 | 0 | 7.1 |
| Frontal pole | 146, 302 | 0 | 0 |
| Temporal pole | 147, 303 | 0 | 50 |
| Transverse temporal | 148, 304 | 0 | 0 |
| Insula | 149-152, 305-308 | 0 | 0 |

For chronic mTBI, inferior temporal, lateral occipital, pars orbitalis, rostral anterior cingulate, frontal pole and temporal pole were selected as the ROI with increased MS. Cuneus, lingual, medial orbitofrontal, parahippocampal, paracentral, pars triangularis, pericalcarine, postcentral, precuneus and superior temporal were selected as the ROIs with decreased MS (**Table S8**). The mean MS of all subregions in each ROI was used to calculate the correlation with cognitive assessments.

**Table S8.** The proportion of altered regions in Desikan-Killiany atlas of chronic mTBI.

| Regions in Desikan Killiany atlas | Label in DKT-308 atlas | Proportion of increased regions (%) | Proportion of decreased regions (%) |
| --- | --- | --- | --- |
| Bankssts | 1-2, 153-154 | 0 | 0 |
| Caudal anterior cingulate | 3, 155 | 0 | 0 |
| Caudal middle frontal | 4-7, 156-159 | 0 | 0 |
| Cuneus | 8-9, 160-162 | 0 | 40 |
| Entorhinal | 10, 163 | 0 | 0 |
| Fusiform | 11-15, 164-168 | 10 | 0 |
| Inferior parietal | 16-23, 169-178 | 27.8 | 0 |
| Inferior temporal | 24-29, 179-183 | 63.6 | 0 |
| Isthmus cingulate | 30-31, 184-185 | 0 | 0 |
| Lateral occipital | 32-40, 186-194 | 33.3 | 5.6 |
| Lateral orbitofrontal | 41-44, 195-198 | 0 | 12.5 |
| Lingual | 45-50, 199-204 | 0 | 50 |
| Medial orbitofrontal | 51-53, 205-207 | 0 | 33.3 |
| Middle temporal | 54-58, 208-213 | 18.2 | 0 |
| Parahippocampal | 59-60, 214-215 | 0 | 75 |
| Paracentral | 61-63, 216-218 | 0 | 33.3 |
| Pars opercularis | 64-66, 219-221 | 0 | 16.7 |
| Pars orbitalis | 67, 222 | 50 | 0 |
| Pars triangularis | 68-69, 223-225 | 0 | 40 |
| Pericalcarine | 70-71, 226-228 | 0 | 40 |
| Postcentral | 72-79, 229-236 | 0 | 43.8 |
| Posterior cingulate | 80-81, 237-238 | 25 | 0 |
| Precentral | 82-90, 239-247 | 0 | 11.1 |
| Precuneus | 91-97, 248-254 | 0 | 35.7 |
| Rostral anterior cingulate | 98, 255 | 100 | 0 |
| Rostral middle frontal | 99-108, 256-265 | 15 | 10 |
| Superior frontal | 109-121, 266-278 | 0 | 11.5 |
| Superior parietal | 122-131, 279-288 | 5 | 10 |
| Superior temporal | 132-138, 289-294 | 7.7 | 38.5 |
| Supra marginal | 139-145, 295-301 | 7.1 | 21.4 |
| Frontal pole | 146, 302 | 50 | 0 |
| Temporal pole | 147, 303 | 100 | 0 |
| Transverse temporal | 148, 304 | 0 | 0 |
| Insula | 149-152, 305-308 | 0 | 25 |

From all the ROI in the chronic phase, there was only a marginal significant correlation between the mean MS of the inferior temporal cortex and the Verbal Fluency Test (VFT) (*r* = -0.343, *p* = 0.055, FDR corrected [*p* = 0.011 before FDR correction], **Figure S3**).


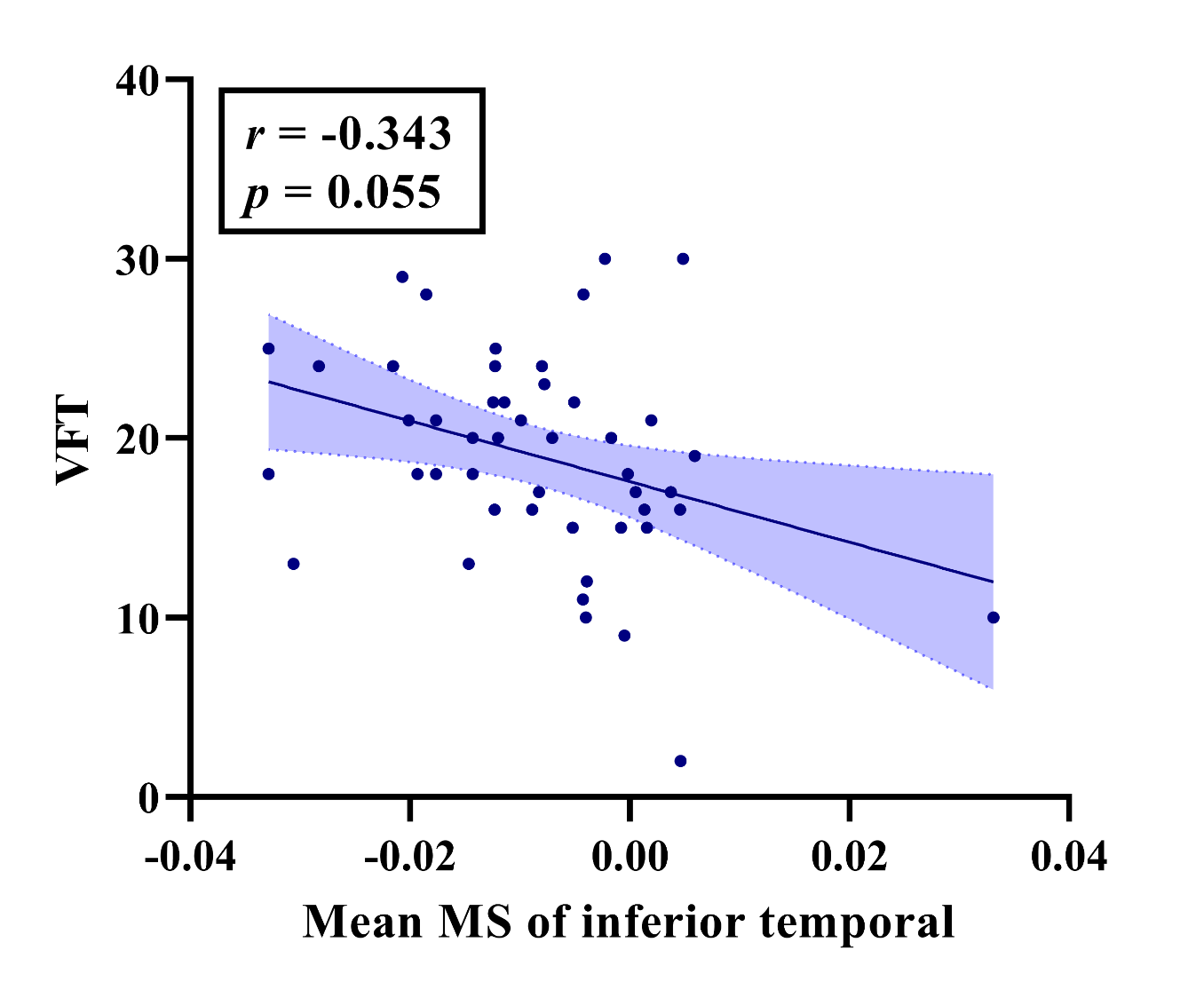


**Figure S3.** The marginal significant negative correlation between MS of the inferior temporal cortex and Verbal Fluency Test (VFT).

**S06: Determine the regions whose MS were correlated with cognition**

We used Partial least squares (PLS) regression ^[1]^ to identify regions whose MS (112 patients × the nodal degrees of MSN in 308 regions with 10% connection density as predictor variables) were significantly correlated with cognitive function (112 patients × 5 neuropsychological assessments including TMT-A, DSC, FDS, BDS and VFT as response variables). The first two components of the PLS (PLS1 and PLS2) explained 40.4% of the variance. PLS1 score was correlated with all the 5 neuropsychological assessments (TMT-A: *r* = 0.379, *p* < 0.0001; DSC: *r* = -0.706, *p* < 0.0001; FDS: *r* = -0.614, *p* < 0.0001; BDS: *r* = -0.471, *p* < 0.0001; VFT: *r* = -0.439, *p* < 0.0001) (**Figure S4**). Furthermore, we investigated the regions in PLS1 whose MS were significantly correlated with cognitive performances by calculating the weight representing the degree of contribution of each region. The positive weights were mostly found in the left hemisphere while the negative weights were mostly observed in the right hemisphere (**Figure S5A**). We finally found 14 significant regions survived after Bonferroni correction which were concentrated in association cortex (**Figure S5B, Table S9**).

**
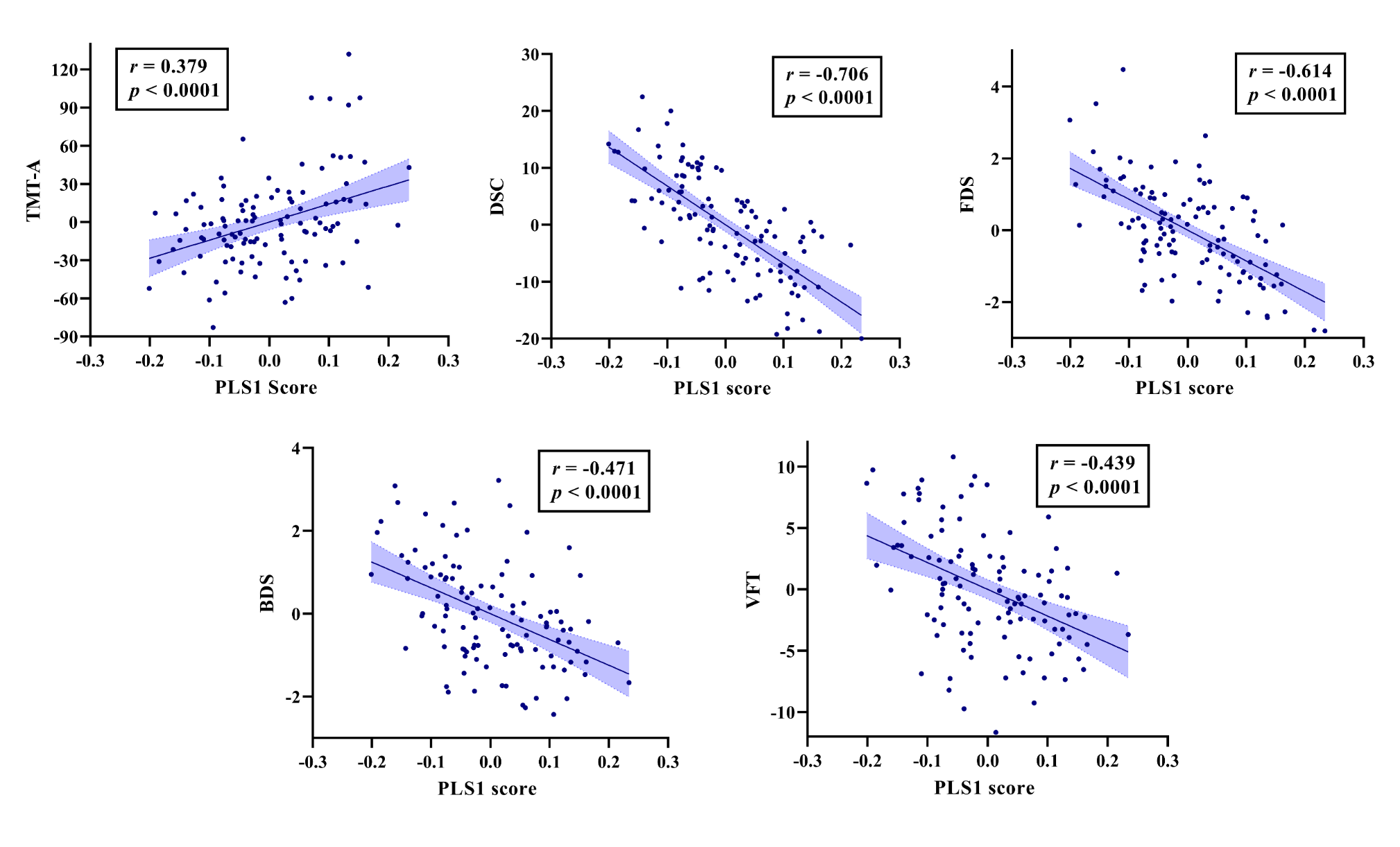
**

**Figure S4.** Correlation between PLS1 score and neuropsychological assessments. The PLS1 score was positively correlated with TMT-A, and negatively correlated with DSC, FDS, BDS and VFT. TMT-A, trail making A test; DSC, digit symbol coding score; FDS, forward digit span; BDS, backward digit span; VFT, verbal fluency test.

**
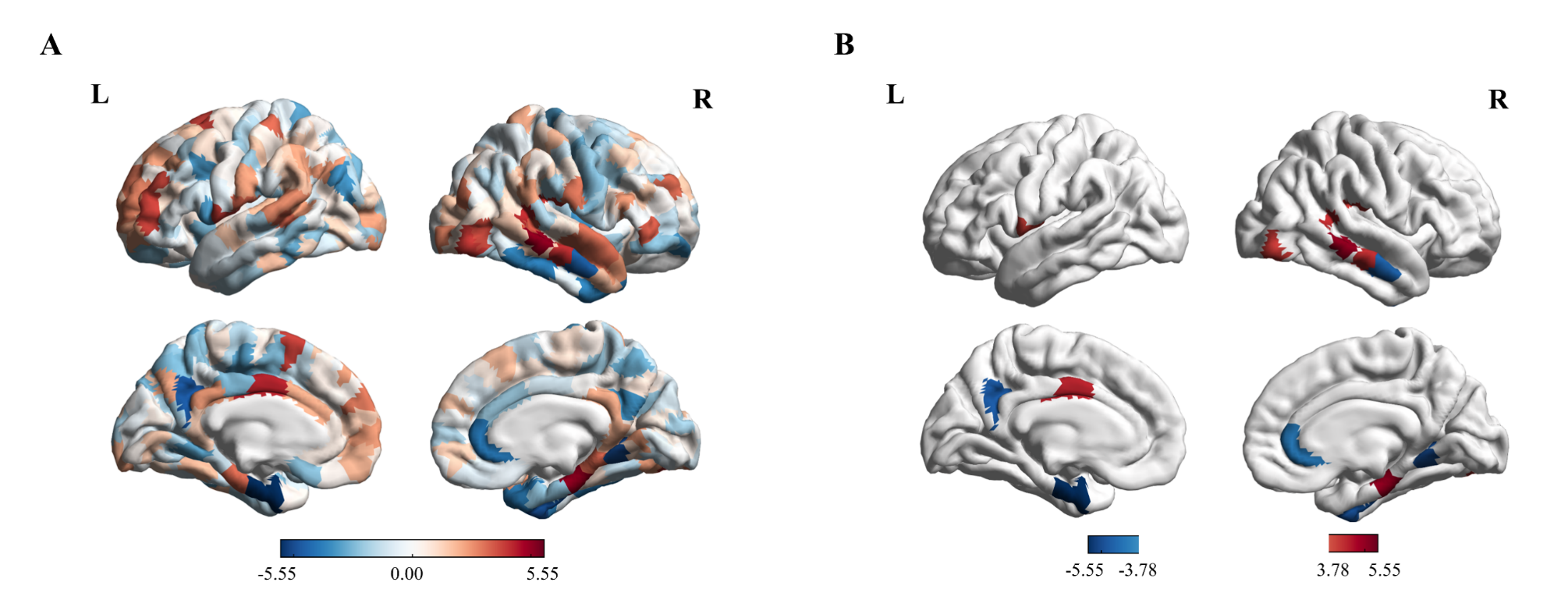
**

**Figure S5.** The contribution of each region to cognitive function. A) The PLS1 weighted rank map of each brain region. B) The significant regions in the rank map.

**Table S9.** The regions whose MS had significant contributions to cognitive function.

| Regions | MNI Coordinates | | | Z-value | P-value  (Bonferroni) |
| --- | --- | --- | --- | --- | --- |
|  | X | Y | Z |  |  |
| rh_middletemporal_part1 | 60.75 | -34.05 | -8.07 | 5.02 | 0.0002 |
| rh_parahippocampal_part2 | 27.45 | -24.86 | -24.20 | 4.92 | 0.0003 |
| lh_posteriorcingulate_part2 | -4.38 | -7.35 | 35.44 | 4.21 | 0.008 |
| rh_bankssts_part2 | 54.99 | -42.90 | 8.95 | 4.12 | 0.012 |
| rh_middletemporal_part6 | 59.99 | -21.62 | -15.33 | 4.05 | 0.016 |
| rh_supramarginal_part2 | 45.03 | -24.93 | 18.41 | 3.91 | 0.029 |
| rh_lateraloccipital_part5 | 43.32 | -78.80 | -11.40 | 3.90 | 0.030 |
| lh_precentral_part2 | -49.77 | 0.86 | 7.72 | 3.78 | 0.048 |
| rh_rostralanteriorcingulate_part1 | 5.98 | 34.53 | 2.54 | -3.87 | 0.034 |
| rh_middletemporal_part3 | 59.99 | -10.80 | -22.33 | -4.48 | 0.002 |
| lh_precuneus_part7 | -7.41 | -56.62 | 28.84 | -4.70 | 0.0008 |
| rh_fusiform_part2 | 34.95 | -7.78 | -36.41 | -4.79 | 0.0005 |
| rh_lingual_part1 | 18.33 | -50.90 | -4.91 | -4.91 | 0.0003 |
| lh_entorhinal_part1 | -24.01 | -5.86 | -32.83 | -5.55 | 8.57E-06 |

**S07: The explanation of hyperdifferentiation, hypercoupling, dedifferentiation and decoupling**

In healthy individuals, regions with positive MS values indicate stronger inter-regional morphological similarity, which corresponds to coupling. If patients exhibit higher MS values in these regions compared to healthy controls, it suggests excessive coupling, or hypercoupling in these regions. Conversely, if patients show lower MS values in these regions, it indicates under-coupling, or decoupling, in these regions. On the other hand, regions with negative MS values in healthy individuals indicate weaker inter-regional morphological similarity, which corresponds to differentiation. If patients exhibit lower MS values in these regions, it suggests excessive differentiation, or hyper-differentiation in these regions. Conversely, if patients show higher MS values in these regions, it indicates under-differentiation, or dedifferentiation, in these regions (**Figure S6**).


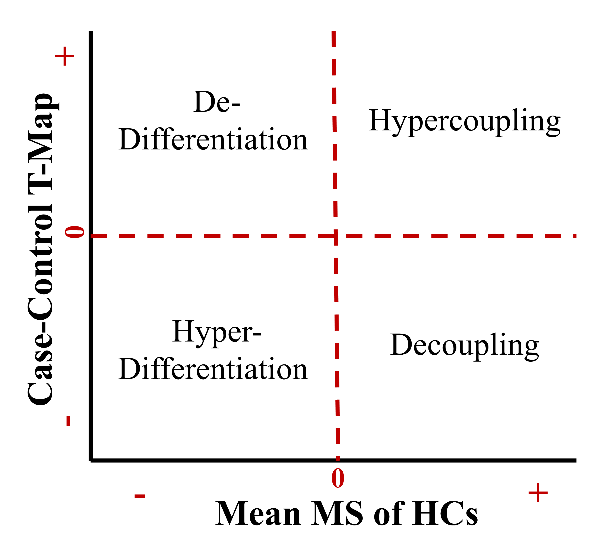


**Figure S6.** Schematic diagram of hypercoupling, hyperdifferentiation, decoupling and dedifferentiation.

**S08: The correlation between Mean MS of HCs and T-Map at baseline for patients with two-time-points data**

The correlation between Mean MS of HCs and T-Map at baseline for the 47 patients with two-time-points data was used to verify the stability of the hypercoupling and hyperdifferentiation results. We found the consistent positive correlation (*r* = 0.469, *p* < 0.0001, **Figure S7**).


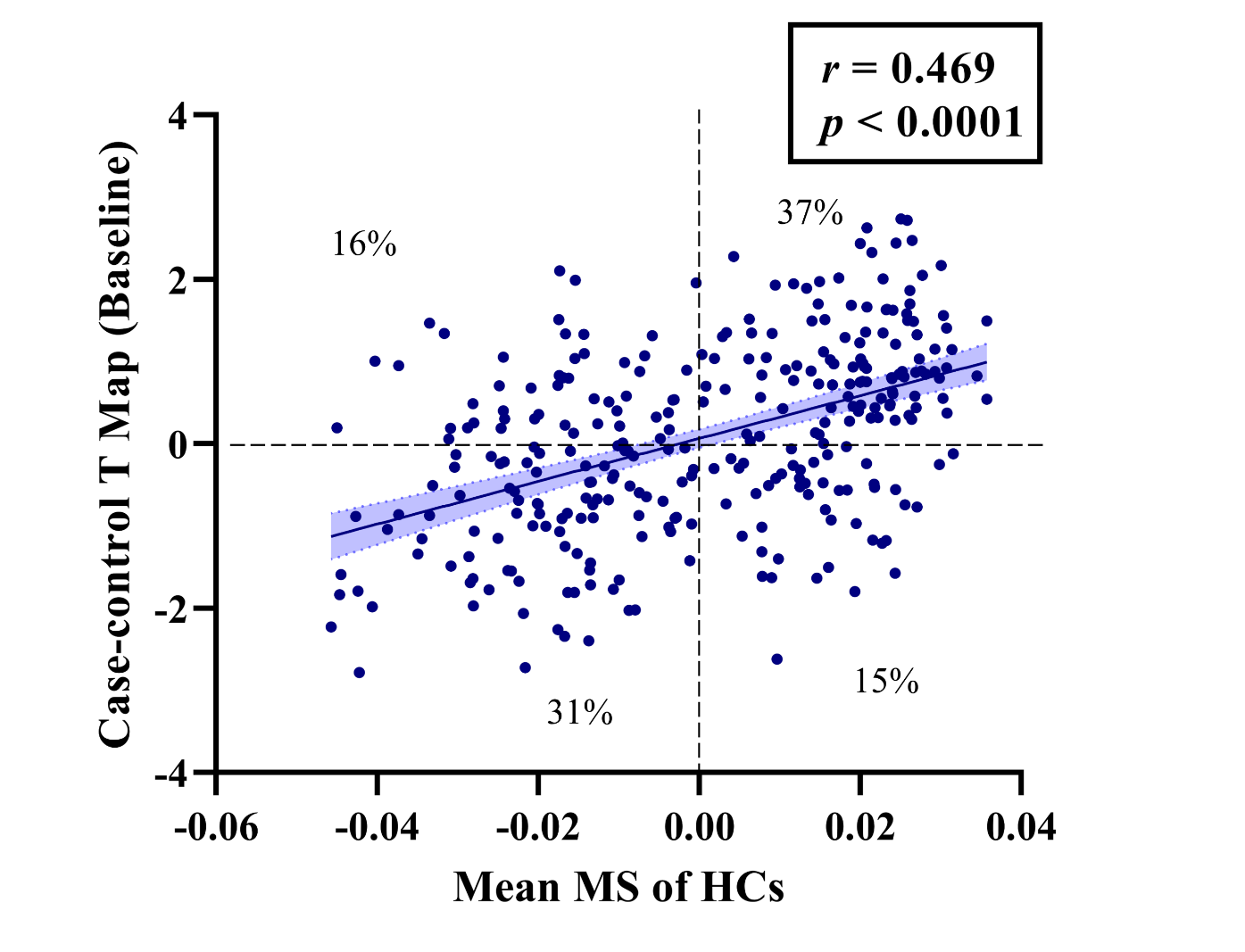


**Figure S7.** The correlation between mean MS of HCs and case-control T-Map at baseline for patients with two-time-points data. Most regions showed hypercoupling (37% of regions) or hyperdifferentiation (31% of regions).

**S09: MS in von Economo 7 cytoarchitectonic classes**

MSN topology has been confirmed to be highly consistent with cortical cytoarchitectonic classes ^[2]^, therefore, we assigned brain regions to von Economo atlas with 7 cytoarchitectonic classes ^[3]^ to observe the altered MS of patients in different cytoarchitectonic classes. Specifically, the seven classes of cytoarchitecture include the agranular cortex corresponding to motor cortices, the frontal cortex and parietal cortex corresponding to association cortices, the cerebral polar cortex corresponding to secondary sensory cortical areas, the granular cortex corresponding to primary sensory cortical areas, as well as the cingulate cortex and insular lobe.

In the acute phase, we found increased MS in the agranular cortex (*p* = 0.012, FDR corrected) and the frontal cortex (*p* = 0.0007, FDR corrected), and decreased MS was observed in the cerebral polar cortex (*p* = 0.0004, FDR corrected) (**Figure S8A**). After dividing patients into three cognitive subgroups (NI, MI and SI), we found increased MS in the agranular cortex in MI subgroup (*p* = 0.020, FDR corrected), increased MS in the frontal cortex in NI (*p* = 0.0099, FDR corrected) and MI (*p* = 0.0099, FDR corrected) subgroup, and decreased MS in the cerebral polar in NI (*p* = 0.0099, FDR corrected) and MI (*p* = 0.006, FDR corrected) subgroup (**Figure S8B**). In the chronic phase, we only found decreased MS in the granular cortex (*p* < 0.0001, FDR corrected) (**Figure S8C**), which mainly occurred in the NI (*p* = 0.005, FDR corrected) and MI (*p* = 0.005, FDR corrected) subgroups (**Figure S8D**).


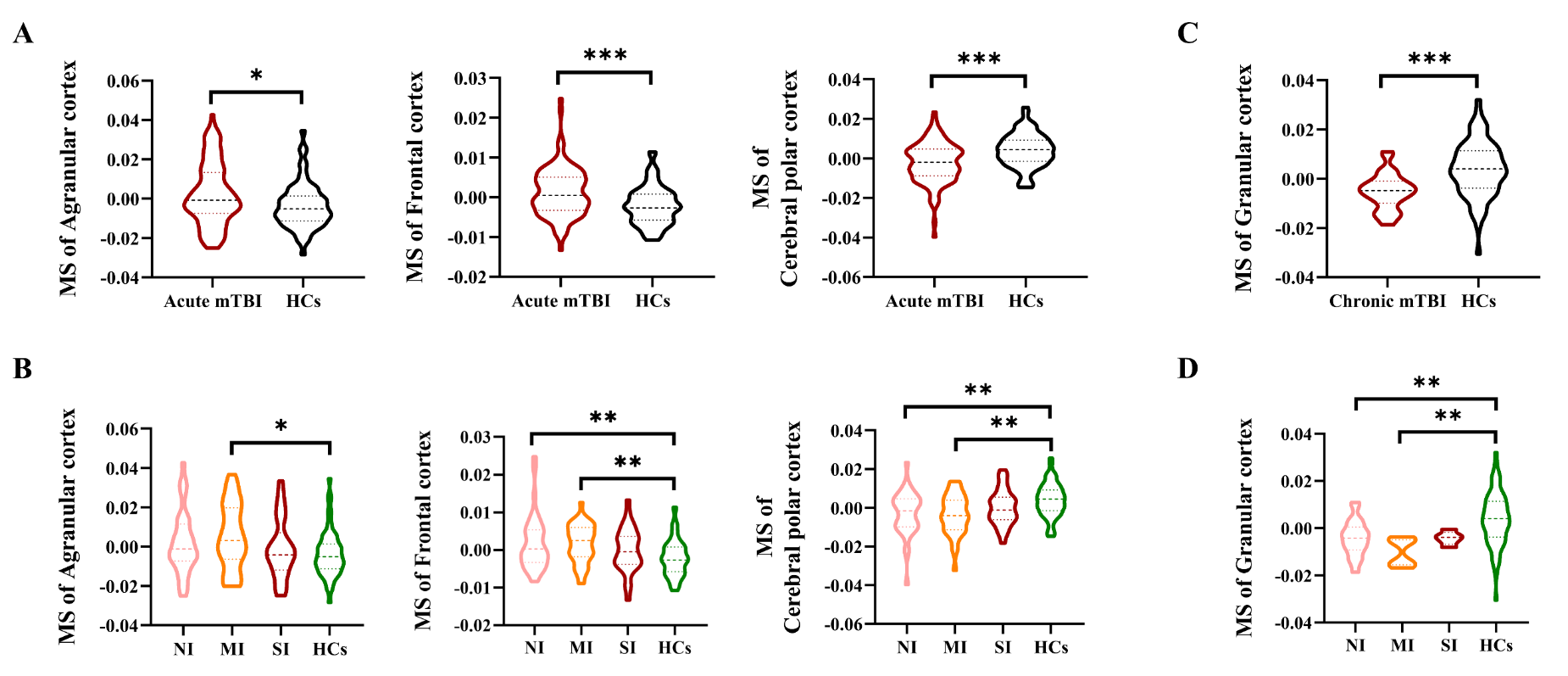


**Figure S8.** MS changes based on cytoarchitectonic classes in patients with mTBI. A) Comparison of significant different MS in the agranular cortex, frontal cortex and cerebral polar cortex between acute mTBI and HCs. B) Among-group comparison of MS based on von Economo atlas in HCs and patients of 3 cognitive subgroups in the acute phase. C) Comparison of significant different MS in the granular cortex between chronic mTBI and HCs. D) Among-group comparison of MS based on von Economo atlas in HCs and patients of 3 cognitive subgroups in the chronic phase. The ∗ indicates the significant difference with FDR-corrected *p* value between 0.01 and 0.05, the ∗∗ displays the FDR-corrected *p* value between 0.001 and 0.01, and the ∗∗∗ represents the FDR-corrected *p* ≤ 0.001.

**S10: MS in Yeo 7 functional networks**

Structural networks have been determined to constrain, maintain, and regulate functional networks, so we assigned brain regions to Yeo atlas with 7 functional networks ^[4]^ to investigate the altered MS of patients in different functional areas. Specifically, the functional networks include the visual (VIS) network, sensorimotor network (SMN), dorsal attention network (DAN), ventral attention network (VAN), limbic (LIM) network, frontoparietal network (FPN) and default mode network (DMN).

In the acute phase, we found increased MS in the SMN (*p* = 0.030, FDR corrected), DAN (*p* = 0.033, FDR corrected) and DMN (*p* = 0.0002, FDR corrected), and decreased MS was observed in the VIS network (*p* = 0.0002, FDR corrected) (**Figure S9A**). After dividing patients into three subgroups, we found increased MS in the DMN in NI (*p* = 0.015, FDR corrected) and MI (*p* = 0.005, FDR corrected) subgroup, and decreased MS in the VIS network in NI (*p* = 0.005, FDR corrected) and MI (*p* = 0.009, FDR corrected) subgroup (**Figure S9B**). In the chronic phase, we only found decreased MS in the SMN (*p* = 0.048, FDR corrected) (**Figure S9C**).


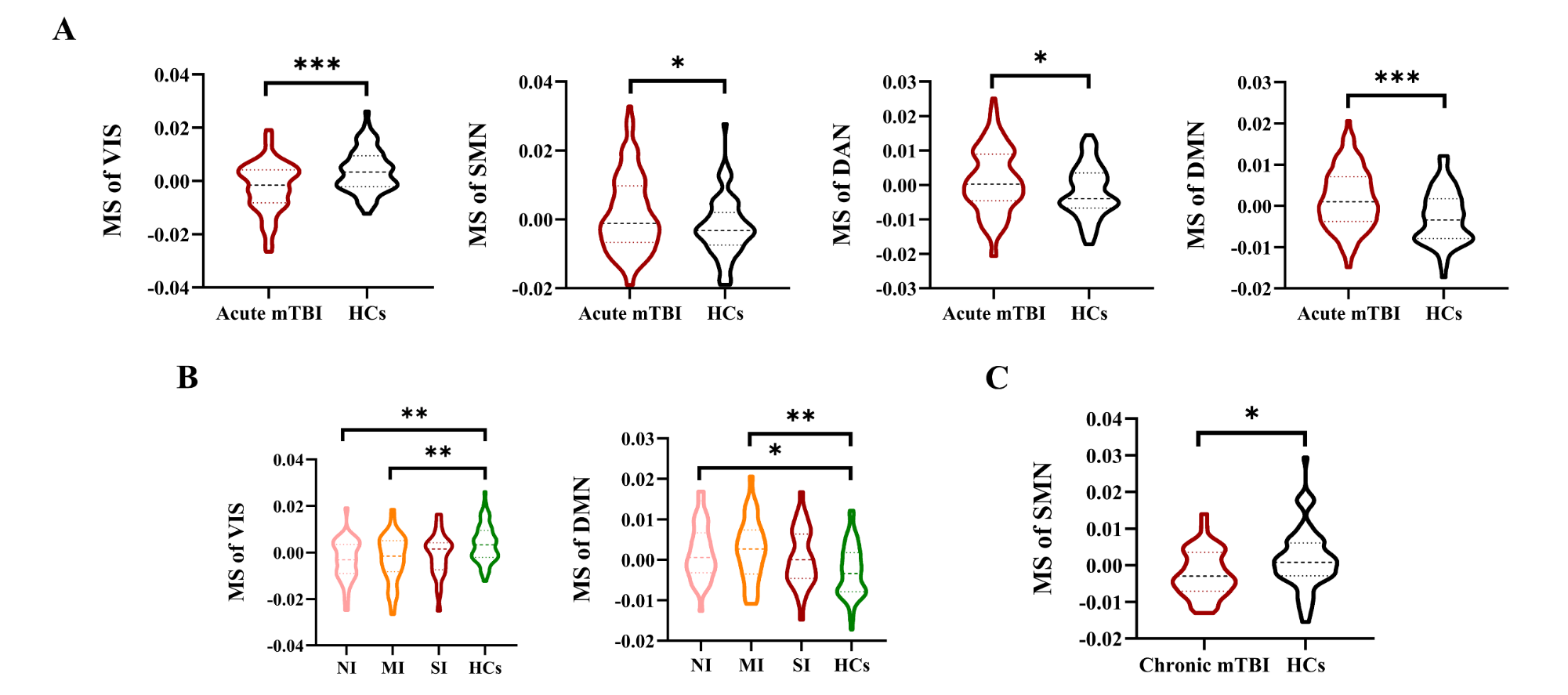


**Figure S9.** MS changes based on functional networks in patients with mTBI. A) Comparison of significant different MS in the VIS network, SMN, DAN and DMN between acute mTBI and HCs. B) Among-group comparison of MS based on Yeo atlas in HCs and patients of 3 cognitive subgroups in the acute phase. C) Comparison of significant different MS in the SMN between chronic mTBI and HCs. The ∗ indicates the significant difference with FDR-corrected *p* value between 0.01 and 0.05, the ∗∗ displays the FDR-corrected *p* value between 0.001 and 0.01, and the ∗∗∗ represents the FDR-corrected *p* ≤ 0.001. VIS, visual; SMN, sensorimotor network; DAN, dorsal attention network; DMN, default mode network.

**S11: Verify the consistency within the control group of pediatric mTBI dataset**

Due to the different sources of control data in pediatric mTBI dataset, we should complete the intra-group comparison to verify the consistency of MS within the control group. Thirty-one subjects were divided randomly into two groups (16 subjects in group1 as test group and 15 subjects in group2 as replicated group). The results showed there were no intra-group differences both in global MS (*p* = 0.88, **Figure S10A**) and in the mean regional MS of 308 regions (*p* = 0.89, **Figure S10B**).

**
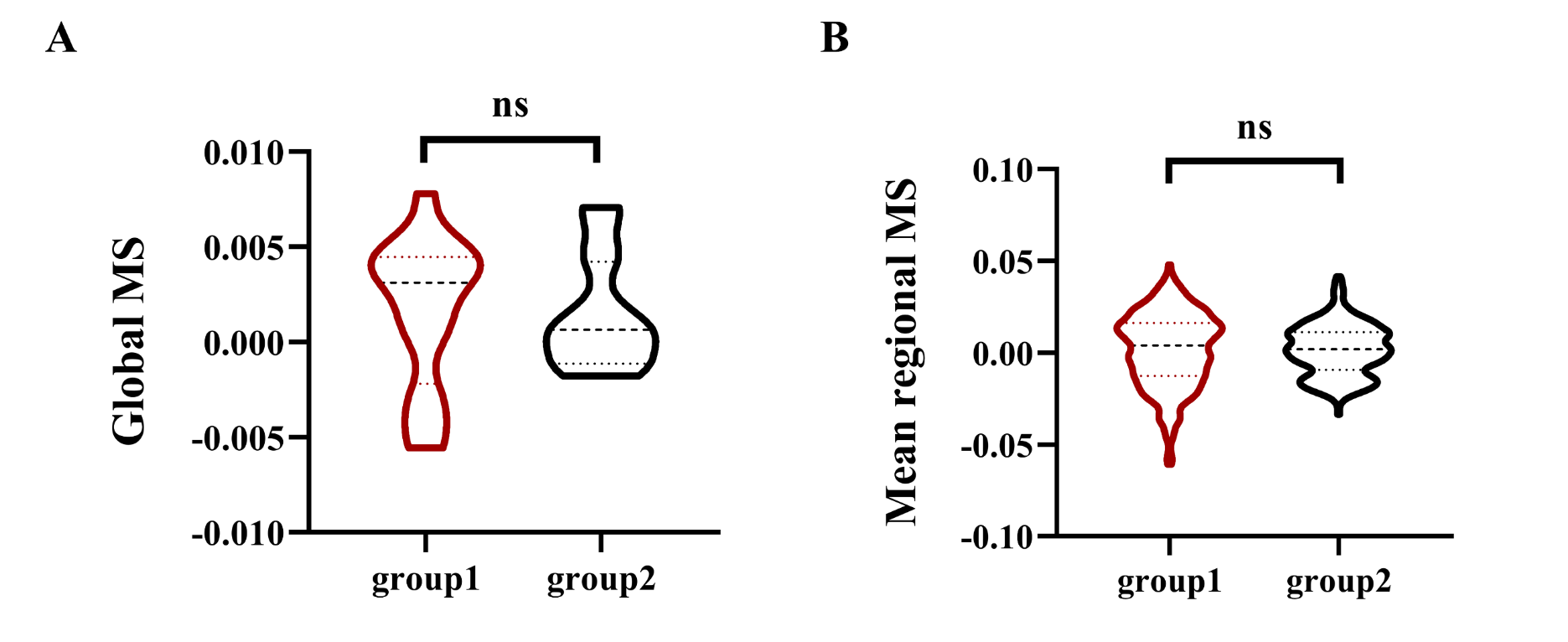
**

**Figure S10.** The intra-group comparison of MS. A) The undifferentiated intra-group global MS of control data. B) The undifferentiated intra-group mean regional MS of control data.

**S12: Altered global MS and regional MS in pediatric mTBI**

**
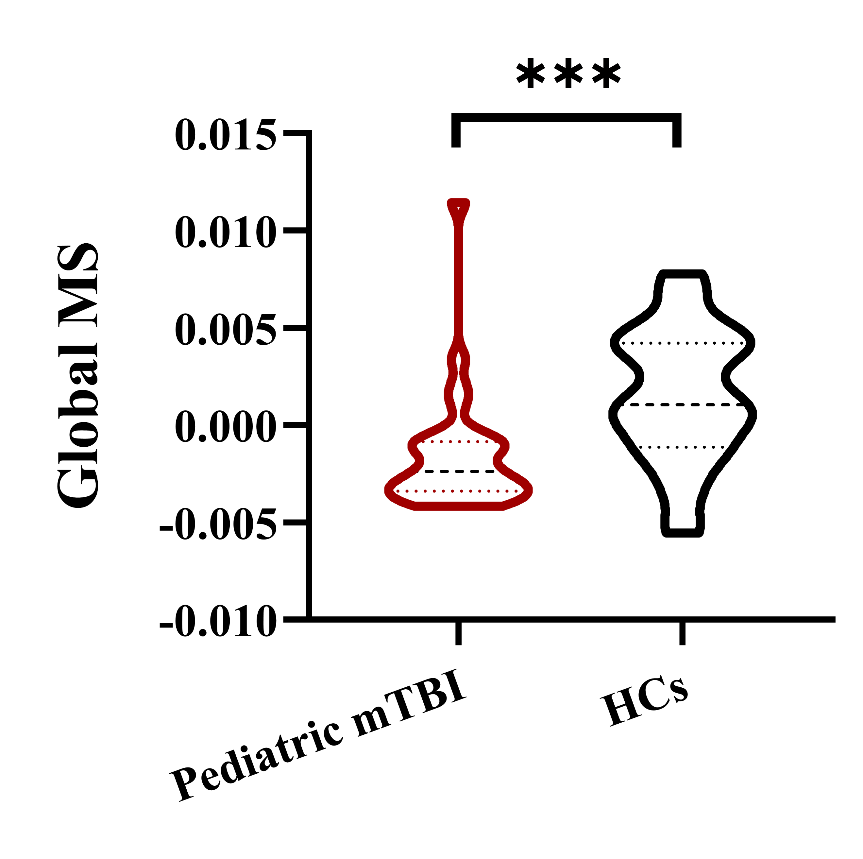
**

**Figure S11.** The between-group differences of global MS. The global MS in children with mTBI decreased significantly. The ∗∗∗ represents the *p* ≤ 0.001.

**
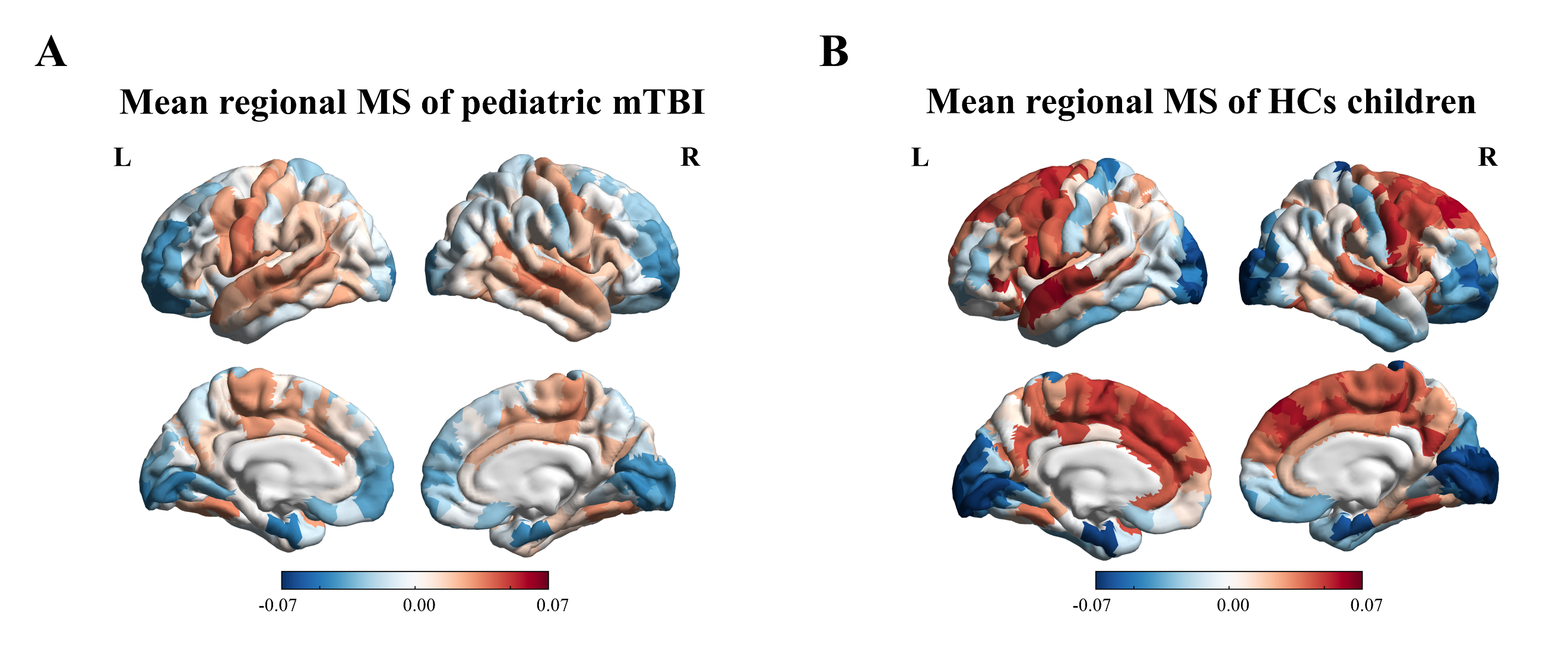
**

**Figure S12.** The regional MS. A) The mean regional MS of pediatric mTBI. B) The mean regional MS of HCs children.

Almost half of the brain regions represented altered MS in pediatric mTBI (147 regions after FDR correction). The 40 regions with the most significant increased and decreased MS are shown in **Table S10**, indicating the strongest alteration of MS in the lateral occipital lobe and superior frontal lobe.

**Table S10.** The regions with the most significant difference of regional MS between children with mTBI and HCs children.

| Regions | MNI Coordinates | | | T-value | | *P* value  (FDR) |
| --- | --- | --- | --- | --- | --- | --- |
|  | X | Y | Z |  |  | |
| lh_lateraloccipital_part1 | -24.91 | -92.30 | 14.19 | 6.513 | 7.91E-07 | |
| lh_postcentral_part5 | -40.49 | -29.29 | 58.13 | 5.724 | 6.07E-06 | |
| rh_lateraloccipital_part8 | 29.43 | -94.00 | -5.36 | 5.363 | 2.03E-05 | |
| rh_lateraloccipital_part4 | 24.05 | -90.63 | 16.78 | 5.331 | 2.16E-05 | |
| lh_superiorparietal_part2 | -12.00 | -89.21 | 25.05 | 5.208 | 3.26E-05 | |
| rh_postcentral_part1 | 13.79 | -34.57 | 71.67 | 5.16 | 3.74E-05 | |
| rh_lateraloccipital_part3 | 17.11 | -99.07 | 10.07 | 5.017 | 5.84E-05 | |
| lh_lateraloccipital_part8 | -24.47 | -93.36 | -13.93 | 4.941 | 7.43E-05 | |
| rh_lateraloccipital_part1 | 18.65 | -99.16 | -7.39 | 4.901 | 8.29E-05 | |
| lh_lateraloccipital_part6 | -34.84 | -84.61 | -14.47 | 4.859 | 9.31E-05 | |
| rh_superiorparietal_part3 | 15.73 | -85.23 | 34.59 | 4.649 | 0.0002 | |
| rh_parstriangularis_part1 | 48.34 | 35.37 | -0.69 | 4.648 | 0.0002 | |
| rh_postcentral_part7 | 43.57 | -23.45 | 53.87 | 4.603 | 0.0002 | |
| rh_lateraloccipital_part9 | 41.60 | -84.25 | -1.24 | 4.535 | 0.0002 | |
| lh_lateraloccipital_part5 | -16.53 | -100.15 | -6.05 | 4.314 | 0.0004 | |
| lh_lateraloccipital_part3 | -14.74 | -99.83 | 8.84 | 4.274 | 0.0005 | |
| rh_lingual_part2 | 8.20 | -89.14 | -8.57 | 4.24 | 0.0005 | |
| lh_lateraloccipital_part7 | -28.70 | -89.41 | 2.44 | 4.201 | 0.0005 | |
| rh_inferiortemporal_part1 | 43.38 | -4.29 | -40.02 | 4.187 | 0.0008 | |
| rh_cuneus_part2 | 6.20 | -89.29 | 13.00 | 4.157 | 0.0006 | |
| lh_caudalmiddlefrontal_part2 | -31.96 | 2.15 | 51.27 | -5.06 | 5.19E-05 | |
| lh_superiorfrontal_part8 | -10.01 | 10.76 | 61.43 | -5.418 | 2.03E-05 | |
| lh_superiorfrontal_part9 | -12.80 | 41.99 | 41.76 | -5.559 | 1.06E-05 | |
| lh_superiorfrontal_part5 | -10.35 | 54.69 | 27.23 | -5.72 | 6.07E-06 | |
| lh_superiorfrontal_part1 | -11.06 | 61.80 | 6.30 | -5.741 | 6.07E-06 | |
| rh_superiorfrontal_part11 | 9.20 | 24.39 | 53.69 | -5.772 | 5.93E-06 | |
| rh_superiorfrontal_part13 | 9.66 | 30.31 | 45.21 | -5.9 | 3.89E-06 | |
| lh_superiorfrontal_part4 | -17.93 | 1.24 | 62.20 | -6.003 | 2.82E-06 | |
| rh_superiorfrontal_part4 | 10.09 | 52.88 | 14.41 | -6.004 | 2.82E-06 | |
| rh_superiorfrontal_part8 | 11.49 | 46.99 | 37.34 | -6.1 | 2.26E-06 | |
| lh_medialorbitofrontal_part2 | -6.50 | 52.61 | -11.10 | -6.145 | 2.08E-06 | |
| lh_superiorfrontal_part11 | -17.72 | 30.84 | 48.03 | -6.374 | 9.48E-07 | |
| rh_superiorfrontal_part10 | 9.11 | 36.26 | 30.57 | -6.382 | 9.48E-07 | |
| rh_rostralmiddlefrontal_part3 | 31.82 | 29.86 | 37.90 | -6.415 | 9.48E-07 | |
| rh_superiorfrontal_part6 | 10.21 | 54.91 | 26.16 | -6.643 | 5.56E-07 | |
| rh_superiorfrontal_part9 | 19.63 | 17.78 | 54.75 | -7.102 | 1.12E-07 | |
| lh_superiorfrontal_part12 | -17.76 | 21.07 | 55.61 | -7.865 | 7.14E-09 | |
| lh_superiorfrontal_part3 | -9.02 | 52.78 | 15.42 | -8.607 | 5.33E-10 | |
| rh_superiorfrontal_part5 | 19.31 | 3.41 | 60.36 | -9.62 | 1.66E-11 | |
| rh_superiorfrontal_part12 | 16.00 | 36.52 | 45.57 | -10.539 | 1.07E-12 | |

lh, left hemisphere; rh, right hemisphere. T-values were obtained from regional MS data (regressed age, sex, and years of education) using two-sample t-tests. All *p* values were adjusted by FDR correction, and were determined based on 2-sided tests.

**S13: MS in von Economo 7 cytoarchitectonic classes and Yeo 7 functional networks of pediatric mTBI**

Compared with HCs children, the increased MS in the cerebral polar cortex (*p* < 0.0001, FDR corrected) and granular cortex (*p* < 0.0001, FDR corrected), and the decreased MS in the agranular cortex (*p* = 0.046, FDR corrected), frontal cortex (*p* < 0.0001, FDR corrected) and parietal cortex (*p* = 0.019, FDR corrected) were found in pediatric mTBI based on cytoarchitectonic classes (**Figure S13A**). For functional networks, the increased MS in the VIS (*p* < 0.0001, FDR corrected), and the decreased MS in the DAN (*p* = 0.0007, FDR corrected), VAN (*p* = 0.0003, FDR corrected), FPN (*p* < 0.0001, FDR corrected) and DMN (*p* < 0.0001, FDR corrected) were found in patients (**Figure S13B**).

**
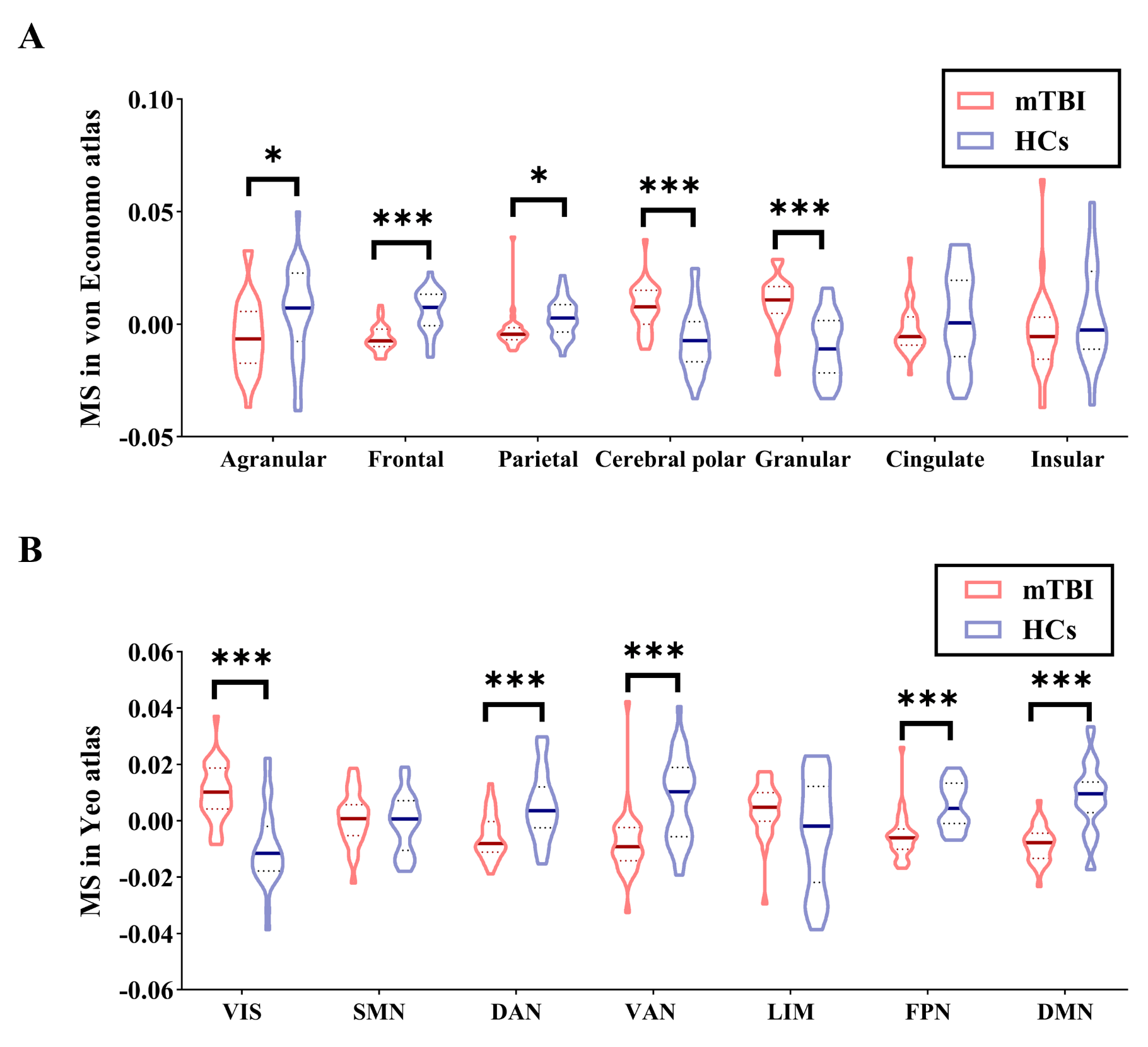
**

**Figure S13.** MS changes based on cytoarchitectonic classes and functional networks in pediatric mTBI. A) Comparison of significant different regional MS in agranular cortex, frontal cortex, parietal cortex, cerebral polar cortex and granular cortex between mTBI and HCs children. B) Comparison of significant different regional MS in VIS network, DAN, VAN, FPN and DMN between mTBI and HCs children. The ∗ indicates the significant difference with FDR-corrected *p* value between 0.01 and 0.05 and the ∗∗∗ represents the FDR-corrected *p* ≤ 0.001. VIS, visual; SMN, sensorimotor network; DAN, dorsal attention network; VAN, ventral attention network; LIM, limbic; FPN, frontoparietal network; DMN, default mode network.

**S14: MTBI-related DEGs**

The volcano plot of up-regulated DEGs and down-regulated DEGs is shown in **Figure S14**. Values of |log_2_ Fold Change (log_2_FC)| > 1 and FDR adjusted *p* < 0.05 (-log_10_*P*_adj_ > 1) were set as the thresholds.

**
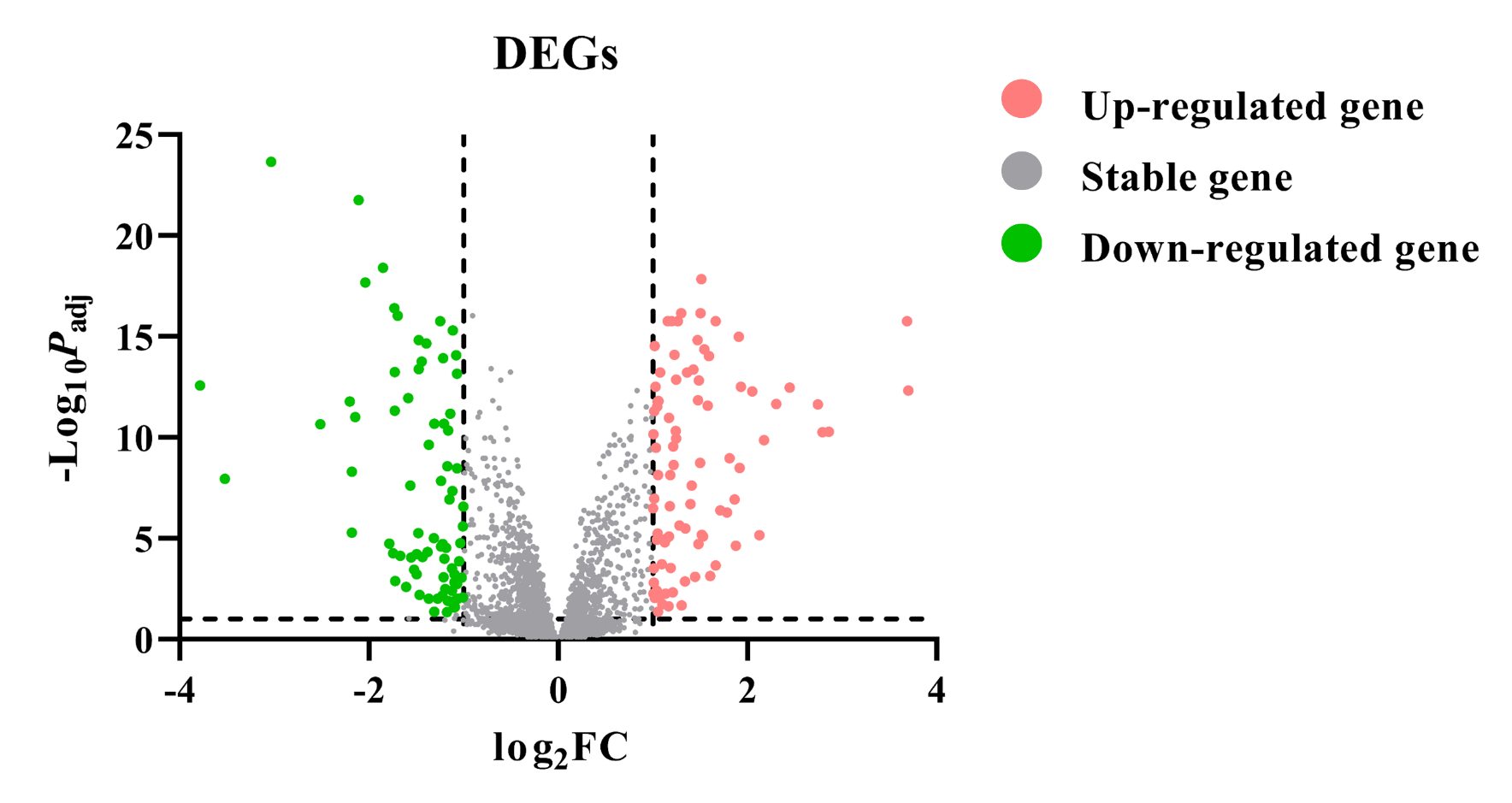
**

**Figure S14. MTBI-related DEG.** The volcano plot shows 77 up-regulated genes (red) and 63 down-regulated genes (blue). DEGs, differentially expressed genes; FC, Fold Change; *P*_adj_, FDR adjusted *p* value.

**S15: Enrichment of PLS gene sets in TBI-related DEGs**

The significant overlapped genes were found between PLS+ genes and down-regulated DEGs (*p* = 0.034, Bonferroni corrected). However, there were no significant overlap between PLS+ genes and up-regulated DEGs (*p* = 1, Bonferroni corrected). In addition, PLS- genes were not enriched in up-regulated (*p* = 0.849, Bonferroni corrected) or down-regulated DEGs (*p* = 1, Bonferroni corrected) (**Table S11**).

**Table S11.** The overlapped genes between PLS1 gene sets and DEGs.

| DEGs Type | PLS type | Overlap | DEGs genes | Gene ratio | *P*_BONF_ value |
| --- | --- | --- | --- | --- | --- |
| Up-regulated | PLS+ | 4 | 77 | 0.052 | 1 |
| Up-regulated | PLS- | 11 | 77 | 0.143 | 0.849 |
| Down-regulated | PLS+ | 8 | 63 | 0.127 | 0.034 |
| Down-regulated | PLS- | 4 | 63 | 0.063 | 1 |

*p* values were adjusted by Bonferroni correction, and were determined based on 2-sided tests.

**S16: Cell-Type Specificity of PLS1 gene set**

The lists of human genes expressed in nine specific cell types including ependymal (Epend), oligodendrocytes (Oligo), microglia (Micro), CA1 pyramidal neurons (CA1), interneurons (Inter), endothelial (Endo), S1 pyramidal neurons (S1), astrocytes (Astro), and mural. The significance of the overlap between PLS1+ / PLS- gene set and cell-type-specific genes are shown in **TableS12** and **TableS13**.

**Table S12.** The significance of the overlap between PLS1+ gene set and cell-type-specific genes.

| Cell Type | Overlapped genes | Cell-type-specific genes | Gene ratio | | *P*_BONF_ value |
| --- | --- | --- | --- | --- | --- |
| Epend | 15 | 391 | 0.038 | 1 | |
| Oligo | 10 | 393 | 0.025 | 1 | |
| Micro | 11 | 382 | 0.029 | 1 | |
| CA1 | 30 | 357 | 0.084 | 0.011 | |
| Inter | 17 | 325 | 0.052 | 1 | |
| Endo | 0 | 321 | 0.000 | 1 | |
| S1 | 12 | 236 | 0.051 | 1 | |
| Astro | 7 | 214 | 0.033 | 1 | |
| Mural | 7 | 134 | 0.052 | 1 | |

Epend, ependymal; Oligo, oligodendrocytes; Micro, microglia; CA1, CA1 pyramidal neurons; Inter, interneurons; Endo, endothelial; S1, S1 pyramidal neurons; Astro, astrocytes. *p* values were adjusted by Bonferroni correction, and were determined based on 2-sided tests.

**Table S13.** The significance of the overlap between PLS1- gene set and cell-type-specific genes.

| Cell Type | Overlapped genes | Cell-type-specific genes | Gene ratio | | *P*_BONF_ value |
| --- | --- | --- | --- | --- | --- |
| Epend | 34 | 391 | 0.087 | 1 | |
| Oligo | 26 | 393 | 0.066 | 1 | |
| Micro | 13 | 382 | 0.034 | 1 | |
| CA1 | 20 | 357 | 0.056 | 1 | |
| Inter | 28 | 325 | 0.086 | 1 | |
| Endo | 26 | 321 | 0.081 | 1 | |
| S1 | 26 | 236 | 0.110 | 1 | |
| Astro | 11 | 214 | 0.051 | 1 | |
| Mural | 9 | 134 | 0.067 | 1 | |

Epend, ependymal; Oligo, oligodendrocytes; Micro, microglia; CA1, CA1 pyramidal neurons; Inter, interneurons; Endo, endothelial; S1, S1 pyramidal neurons; Astro, astrocytes. *p* values were adjusted by Bonferroni correction, and were determined based on 2-sided tests.

**S17: The association between gene expression and altered regional MS in chronic mTBI**

We found significant positive correlation between PLS1 gene expression map and T-Map (r = 0.320, *p* < 0.0001, **Figure S15A**). However, the first two partial least squares components can only explain 10.21% of the variance (*p* > 0.05). Moreover, neither PLS+ (*r* = 0.059, *p* = 0.472) nor PLS- (*r* = -0.057, *p* = 0.491) had correlation with T-Map (**Figure S15B-C**). Therefore, we determine the uncorrelation between gene expression and MS changes of mTBI patients during the follow-up period.

**
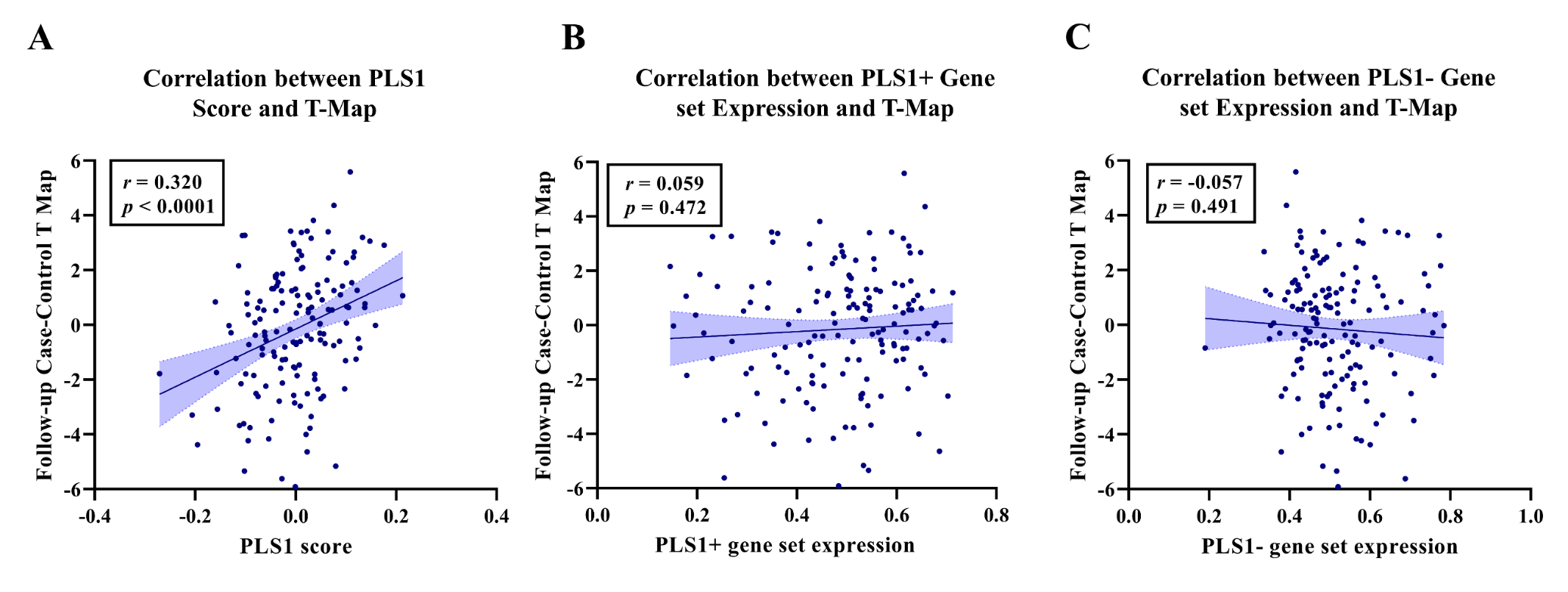
**

**Figure S15.** The association between gene expression and altered regional MS in chronic phase of mTBI. A) The point plot shows the positive correlation between PLS1 gene expression map (PLS1 score) and regional MS T-Map in chronic mTBI. B) The uncorrelation between the expression of the PLS1+ gene set and the regional MS difference. C) The uncorrelation between the expression of the PLS1- gene set and the regional MS difference.

**S18: Experimental design figure and the inclusion and exclusion criteria of adult mTBI dataset**


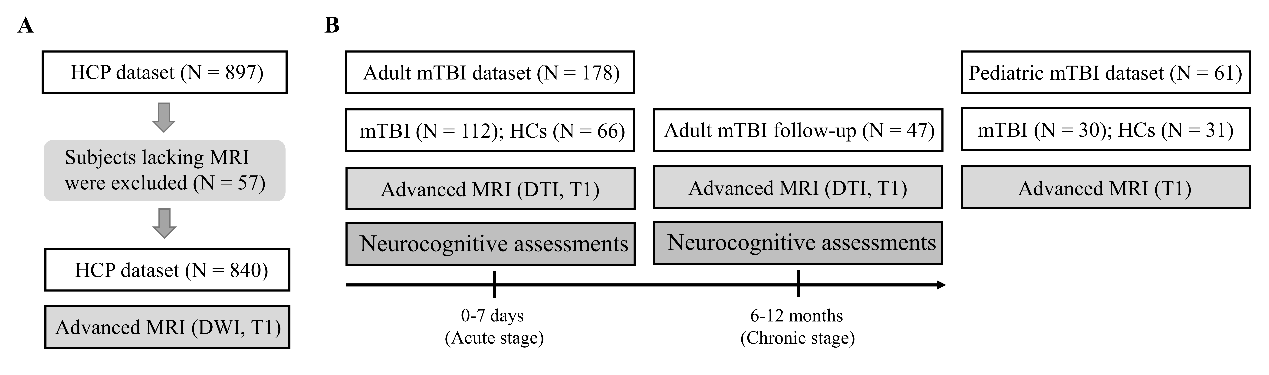


**Figure S16.** Individual assessment protocols, details of the assessment of each type of data, as well as the acquisition and usage processes of A) HCP dataset and B) mTBI dataset.

The inclusion criteria of mTBI were (i) 18 years of age or older, (ii) one or more of the following: confusion or disorientation, loss of consciousness for ≤30 minutes, post-traumatic amnesia for <24 hours, and/or other transient neurological abnormalities (e.g. seizure, focal signs, intracranial lesion not requiring surgery), (iii) initial Glasgow Coma Scale (GCS) score of 13–15, and (iv) diagnosed within 1 week after injury. The exclusion criteria of patients were (i) MRI contraindications, (ii) structural abnormality on conventional neuroimaging, (iii) patients with a history of alcohol or drugs abuse, (iv) patients with a premorbid condition (e.g. previous brain injury history, preexisting headache, or neurological disease), and (v) the manifestations of mTBI due to other injuries (e.g. systemic injuries, facial injuries or spinal cord injury) or other problems (e.g. psychological trauma, language barrier or coexisting medical conditions. Eligibility criteria for healthy control participants included a lack of any contraindications to MRI, neurological impairment, or psychiatric disorders.

**S19: The details of T1w data preprocessing**

The preprocessing of structural MRI data entailed basic image preprocessing, image segmentation, spherical surface construction, brain spherical mapping, and automatic cortical parcellation. All of the steps can be implemented uniformly through the ‘recon-all ‘ command from Freesurfer software. The detailed explanation of all processing steps for the ‘recon-all‘ command is presented in **Table S14**.

**Table S14**. The preprocessing steps implemented by the ‘recon-all‘ command

| Steps | Description |
| --- | --- |
| Step1: Basic image preprocessing | 1. Motion correction and conform |
|  | 1. Non-Uniform intensity normalization |
|  | 1. Talairach transform computation |
|  | 1. Intensity normalization 1 |
|  | 1. Skull strip |
| Step2: Image segmentation, resulting in labeled structural parcellation, as well as spherical surface construction | 1. Linear volumetric registration |
|  | 1. Canonical intensity normalization based on Gaussian Classifier Atlas |
|  | 1. Canonical non-linear volumetric registration |
|  | 1. Remove neck |
|  | 1. Registration with skull |
|  | 1. Volumetric labeling (using Freesurfer standard label, ie Aseg) and statistics |
|  | 1. Intensity normalization 2 (the normalization after removing skull) |
|  | 1. White matter segmentation |
|  | 1. Edit white matter With ASeg |
|  | 1. Create original surface and tessellation |
|  | 1. Orig surface smoothing and inflating (Smooth1 and Inflate 1) |
|  | 1. Automatic topology fixer to obtain suitable surface |
|  | 1. Create final surface (including thickness surface and curve surface) |
|  | 1. Smooth2 and Inflate2 after automatic topology fixer |
| Step3: Brain spherical mapping and automatic cortical parcellation | 1. Spherical mapping (inflate the surface into a sphere) |
|  | 1. Spherical registration (register the surface onto the spherical atlas) |
|  | 1. Map average curvature to subject |
|  | 1. Cortical parcellation - Desikan_Killiany (68 regions) and others (Labeling) |
|  | 1. Cortical parcellation statistics, including structure names, number of vertices, surface area, gray matter volume, average cortical thickness, the standard error of cortical thickness, mean curvature, gaussian curvature, folding index, and intrinsic curvature index. |

**S20: Modules corresponding to DK-308 atlas**

In the 7-module solution with γ = 1.1 and network sparsity thresholds = 10%, the modules containing each region in the DK-308 template are shown in **Table S15**.

**Table S15.** The detailed modular solution in each region.

| Region Name  (Left Hemisphere) | Modular Solution | | Region Name  (Right Hemisphere) | | Modular Solution |
| --- | --- | --- | --- | --- | --- |
| lh_bankssts_part1 | 7 | rh_bankssts_part1 | | 3 | |
| lh_bankssts_part2 | 7 | rh_bankssts_part2 | | 1 | |
| lh_caudalanteriorcingulate_part1 | 1 | rh_caudalanteriorcingulate_part1 | | 5 | |
| lh_caudalmiddlefrontal_part1 | 7 | rh_caudalmiddlefrontal_part1 | | 7 | |
| lh_caudalmiddlefrontal_part2 | 7 | rh_caudalmiddlefrontal_part2 | | 7 | |
| lh_caudalmiddlefrontal_part3 | 5 | rh_caudalmiddlefrontal_part3 | | 5 | |
| lh_caudalmiddlefrontal_part4 | 1 | rh_caudalmiddlefrontal_part4 | | 7 | |
| lh_cuneus_part1 | 3 | rh_cuneus_part1 | | 6 | |
| lh_cuneus_part2 | 3 | rh_cuneus_part2 | | 3 | |
| lh_entorhinal_part1 | 6 | rh_cuneus_part3 | | 3 | |
| lh_fusiform_part1 | 1 | rh_entorhinal_part1 | | 6 | |
| lh_fusiform_part2 | 6 | rh_fusiform_part1 | | 5 | |
| lh_fusiform_part3 | 7 | rh_fusiform_part2 | | 6 | |
| lh_fusiform_part4 | 6 | rh_fusiform_part3 | | 5 | |
| lh_fusiform_part5 | 5 | rh_fusiform_part4 | | 4 | |
| lh_inferiorparietal_part1 | 6 | rh_fusiform_part5 | | 3 | |
| lh_inferiorparietal_part2 | 5 | rh_inferiorparietal_part1 | | 1 | |
| lh_inferiorparietal_part3 | 7 | rh_inferiorparietal_part2 | | 5 | |
| lh_inferiorparietal_part4 | 1 | rh_inferiorparietal_part3 | | 1 | |
| lh_inferiorparietal_part5 | 6 | rh_inferiorparietal_part4 | | 5 | |
| lh_inferiorparietal_part6 | 5 | rh_inferiorparietal_part5 | | 5 | |
| lh_inferiorparietal_part7 | 5 | rh_inferiorparietal_part6 | | 6 | |
| lh_inferiorparietal_part8 | 7 | rh_inferiorparietal_part7 | | 1 | |
| lh_inferiortemporal_part1 | 6 | rh_inferiorparietal_part8 | | 1 | |
| lh_inferiortemporal_part2 | 5 | rh_inferiorparietal_part9 | | 5 | |
| lh_inferiortemporal_part3 | 6 | rh_inferiorparietal_part10 | | 1 | |
| lh_inferiortemporal_part4 | 1 | rh_inferiortemporal_part1 | | 6 | |
| lh_inferiortemporal_part5 | 4 | rh_inferiortemporal_part2 | | 5 | |
| lh_inferiortemporal_part6 | 6 | rh_inferiortemporal_part3 | | 4 | |
| lh_isthmuscingulate_part1 | 3 | rh_inferiortemporal_part4 | | 6 | |
| lh_isthmuscingulate_part2 | 5 | rh_inferiortemporal_part5 | | 4 | |
| lh_lateraloccipital_part1 | 3 | rh_isthmuscingulate_part1 | | 1 | |
| lh_lateraloccipital_part2 | 6 | rh_isthmuscingulate_part2 | | 1 | |
| lh_lateraloccipital_part3 | 3 | rh_lateraloccipital_part1 | | 3 | |
| lh_lateraloccipital_part4 | 5 | rh_lateraloccipital_part2 | | 1 | |
| lh_lateraloccipital_part5 | 3 | rh_lateraloccipital_part3 | | 3 | |
| lh_lateraloccipital_part6 | 7 | rh_lateraloccipital_part4 | | 6 | |
| lh_lateraloccipital_part7 | 6 | rh_lateraloccipital_part5 | | 7 | |
| lh_lateraloccipital_part8 | 3 | rh_lateraloccipital_part6 | | 6 | |
| lh_lateraloccipital_part9 | 3 | rh_lateraloccipital_part7 | | 3 | |
| lh_lateralorbitofrontal_part1 | 4 | rh_lateraloccipital_part8 | | 3 | |
| lh_lateralorbitofrontal_part2 | 4 | rh_lateraloccipital_part9 | | 7 | |
| lh_lateralorbitofrontal_part3 | 4 | rh_lateralorbitofrontal_part1 | | 4 | |
| lh_lateralorbitofrontal_part4 | 6 | rh_lateralorbitofrontal_part2 | | 7 | |
| lh_lingual_part1 | 7 | rh_lateralorbitofrontal_part3 | | 3 | |
| lh_lingual_part2 | 3 | rh_lateralorbitofrontal_part4 | | 4 | |
| lh_lingual_part3 | 7 | rh_lingual_part1 | | 3 | |
| lh_lingual_part4 | 3 | rh_lingual_part2 | | 3 | |
| lh_lingual_part5 | 3 | rh_lingual_part3 | | 7 | |
| lh_lingual_part6 | 3 | rh_lingual_part4 | | 3 | |
| lh_medialorbitofrontal_part1 | 4 | rh_lingual_part5 | | 3 | |
| lh_medialorbitofrontal_part2 | 1 | rh_lingual_part6 | | 3 | |
| lh_medialorbitofrontal_part3 | 4 | rh_medialorbitofrontal_part1 | | 7 | |
| lh_middletemporal_part1 | 6 | rh_medialorbitofrontal_part2 | | 4 | |
| lh_middletemporal_part2 | 5 | rh_medialorbitofrontal_part3 | | 4 | |
| lh_middletemporal_part3 | 6 | rh_middletemporal_part1 | | 3 | |
| lh_middletemporal_part4 | 3 | rh_middletemporal_part2 | | 6 | |
| lh_middletemporal_part5 | 1 | rh_middletemporal_part3 | | 3 | |
| lh_parahippocampal_part1 | 2 | rh_middletemporal_part4 | | 1 | |
| lh_parahippocampal_part2 | 5 | rh_middletemporal_part5 | | 1 | |
| lh_paracentral_part1 | 4 | rh_middletemporal_part6 | | 3 | |
| lh_paracentral_part2 | 7 | rh_parahippocampal_part1 | | 5 | |
| lh_paracentral_part3 | 7 | rh_parahippocampal_part2 | | 2 | |
| lh_parsopercularis_part1 | 7 | rh_paracentral_part1 | | 1 | |
| lh_parsopercularis_part2 | 3 | rh_paracentral_part2 | | 1 | |
| lh_parsopercularis_part3 | 7 | rh_paracentral_part3 | | 1 | |
| lh_parsorbitalis_part1 | 4 | rh_parsopercularis_part1 | | 1 | |
| lh_parstriangularis_part1 | 5 | rh_parsopercularis_part2 | | 5 | |
| lh_parstriangularis_part2 | 5 | rh_parsopercularis_part3 | | 7 | |
| lh_pericalcarine_part1 | 3 | rh_parsorbitalis_part1 | | 5 | |
| lh_pericalcarine_part2 | 3 | rh_parstriangularis_part1 | | 5 | |
| lh_postcentral_part1 | 6 | rh_parstriangularis_part2 | | 1 | |
| lh_postcentral_part2 | 6 | rh_parstriangularis_part3 | | 5 | |
| lh_postcentral_part3 | 6 | rh_pericalcarine_part1 | | 3 | |
| lh_postcentral_part4 | 5 | rh_pericalcarine_part2 | | 3 | |
| lh_postcentral_part5 | 6 | rh_pericalcarine_part3 | | 3 | |
| lh_postcentral_part6 | 6 | rh_postcentral_part1 | | 6 | |
| lh_postcentral_part7 | 6 | rh_postcentral_part2 | | 1 | |
| lh_postcentral_part8 | 6 | rh_postcentral_part3 | | 6 | |
| lh_posteriorcingulate_part1 | 4 | rh_postcentral_part4 | | 5 | |
| lh_posteriorcingulate_part2 | 1 | rh_postcentral_part5 | | 6 | |
| lh_precentral_part1 | 7 | rh_postcentral_part6 | | 6 | |
| lh_precentral_part2 | 1 | rh_postcentral_part7 | | 6 | |
| lh_precentral_part3 | 7 | rh_postcentral_part8 | | 6 | |
| lh_precentral_part4 | 3 | rh_posteriorcingulate_part1 | | 7 | |
| lh_precentral_part5 | 7 | rh_posteriorcingulate_part2 | | 6 | |
| lh_precentral_part6 | 7 | rh_precentral_part1 | | 1 | |
| lh_precentral_part7 | 7 | rh_precentral_part2 | | 7 | |
| lh_precentral_part8 | 7 | rh_precentral_part3 | | 3 | |
| lh_precentral_part9 | 7 | rh_precentral_part4 | | 7 | |
| lh_precuneus_part1 | 7 | rh_precentral_part5 | | 7 | |
| lh_precuneus_part2 | 4 | rh_precentral_part6 | | 7 | |
| lh_precuneus_part3 | 5 | rh_precentral_part7 | | 7 | |
| lh_precuneus_part4 | 7 | rh_precentral_part8 | | 7 | |
| lh_precuneus_part5 | 7 | rh_precentral_part9 | | 7 | |
| lh_precuneus_part6 | 7 | rh_precuneus_part1 | | 5 | |
| lh_precuneus_part7 | 7 | rh_precuneus_part2 | | 5 | |
| lh_rostralanteriorcingulate_part1 | 4 | rh_precuneus_part3 | | 6 | |
| lh_rostralmiddlefrontal_part1 | 5 | rh_precuneus_part4 | | 6 | |
| lh_rostralmiddlefrontal_part2 | 1 | rh_precuneus_part5 | | 5 | |
| lh_rostralmiddlefrontal_part3 | 5 | rh_precuneus_part6 | | 5 | |
| lh_rostralmiddlefrontal_part4 | 7 | rh_precuneus_part7 | | 5 | |
| lh_rostralmiddlefrontal_part5 | 5 | rh_rostralanteriorcingulate_part1 | | 6 | |
| lh_rostralmiddlefrontal_part6 | 1 | rh_rostralmiddlefrontal_part1 | | 7 | |
| lh_rostralmiddlefrontal_part7 | 6 | rh_rostralmiddlefrontal_part2 | | 3 | |
| lh_rostralmiddlefrontal_part8 | 1 | rh_rostralmiddlefrontal_part3 | | 5 | |
| lh_rostralmiddlefrontal_part9 | 5 | rh_rostralmiddlefrontal_part4 | | 7 | |
| lh_rostralmiddlefrontal_part10 | 1 | rh_rostralmiddlefrontal_part5 | | 5 | |
| lh_superiorfrontal_part1 | 7 | rh_rostralmiddlefrontal_part6 | | 7 | |
| lh_superiorfrontal_part2 | 7 | rh_rostralmiddlefrontal_part7 | | 5 | |
| lh_superiorfrontal_part3 | 1 | rh_rostralmiddlefrontal_part8 | | 4 | |
| lh_superiorfrontal_part4 | 1 | rh_rostralmiddlefrontal_part9 | | 7 | |
| lh_superiorfrontal_part5 | 1 | rh_rostralmiddlefrontal_part10 | | 6 | |
| lh_superiorfrontal_part6 | 7 | rh_superiorfrontal_part1 | | 1 | |
| lh_superiorfrontal_part7 | 1 | rh_superiorfrontal_part2 | | 7 | |
| lh_superiorfrontal_part8 | 3 | rh_superiorfrontal_part3 | | 2 | |
| lh_superiorfrontal_part9 | 7 | rh_superiorfrontal_part4 | | 1 | |
| lh_superiorfrontal_part10 | 7 | rh_superiorfrontal_part5 | | 7 | |
| lh_superiorfrontal_part11 | 1 | rh_superiorfrontal_part6 | | 1 | |
| lh_superiorfrontal_part12 | 1 | rh_superiorfrontal_part7 | | 1 | |
| lh_superiorfrontal_part13 | 3 | rh_superiorfrontal_part8 | | 1 | |
| lh_superiorparietal_part1 | 6 | rh_superiorfrontal_part9 | | 7 | |
| lh_superiorparietal_part2 | 6 | rh_superiorfrontal_part10 | | 1 | |
| lh_superiorparietal_part3 | 6 | rh_superiorfrontal_part11 | | 1 | |
| lh_superiorparietal_part4 | 6 | rh_superiorfrontal_part12 | | 7 | |
| lh_superiorparietal_part5 | 6 | rh_superiorfrontal_part13 | | 1 | |
| lh_superiorparietal_part6 | 6 | rh_superiorparietal_part1 | | 6 | |
| lh_superiorparietal_part7 | 6 | rh_superiorparietal_part2 | | 6 | |
| lh_superiorparietal_part8 | 6 | rh_superiorparietal_part3 | | 6 | |
| lh_superiorparietal_part9 | 6 | rh_superiorparietal_part4 | | 6 | |
| lh_superiorparietal_part10 | 3 | rh_superiorparietal_part5 | | 6 | |
| lh_superiortemporal_part1 | 7 | rh_superiorparietal_part6 | | 6 | |
| lh_superiortemporal_part2 | 1 | rh_superiorparietal_part7 | | 4 | |
| lh_superiortemporal_part3 | 3 | rh_superiorparietal_part8 | | 6 | |
| lh_superiortemporal_part4 | 1 | rh_superiorparietal_part9 | | 6 | |
| lh_superiortemporal_part5 | 3 | rh_superiorparietal_part10 | | 6 | |
| lh_superiortemporal_part6 | 3 | rh_superiortemporal_part1 | | 1 | |
| lh_superiortemporal_part7 | 1 | rh_superiortemporal_part2 | | 3 | |
| lh_supramarginal_part1 | 6 | rh_superiortemporal_part3 | | 1 | |
| lh_supramarginal_part2 | 5 | rh_superiortemporal_part4 | | 1 | |
| lh_supramarginal_part3 | 5 | rh_superiortemporal_part5 | | 3 | |
| lh_supramarginal_part4 | 5 | rh_superiortemporal_part6 | | 3 | |
| lh_supramarginal_part5 | 5 | rh_supramarginal_part1 | | 6 | |
| lh_supramarginal_part6 | 5 | rh_supramarginal_part2 | | 6 | |
| lh_supramarginal_part7 | 6 | rh_supramarginal_part3 | | 5 | |
| lh_frontalpole_part1 | 5 | rh_supramarginal_part4 | | 1 | |
| lh_temporalpole_part1 | 6 | rh_supramarginal_part5 | | 1 | |
| lh_transversetemporal_part1 | 5 | rh_supramarginal_part6 | | 1 | |
| lh_insula_part1 | 7 | rh_supramarginal_part7 | | 7 | |
| lh_insula_part2 | 2 | rh_frontalpole_part1 | | 5 | |
| lh_insula_part3 | 2 | rh_temporalpole_part1 | | 6 | |
| lh_insula_part4 | 3 | rh_transversetemporal_part1 | | 5 | |
|  |  | rh_insula_part1 | | 2 | |
|  |  | rh_insula_part2 | | 5 | |
|  |  | rh_insula_part3 | | 2 | |
|  |  | rh_insula_part4 | | 2 | |

**References**

[1] H. Abdi, L. J. Williams, *Methods Mol Biol* **2013**, *930*, 549.

[2] J. Seidlitz, F. Váša, M. Shinn, R. Romero-Garcia, K. J. Whitaker, P. E. Vértes, K. Wagstyl, P. Kirkpatrick Reardon, L. Clasen, S. Liu, A. Messinger, D. A. Leopold, P. Fonagy, R. J. Dolan, P. B. Jones, I. M. Goodyer, A. Raznahan, E. T. Bullmore, *Neuron* **2018**, *97* (1), 231.

[3] a) *Archives of Neurology And Psychiatry* **1926**, *16* (6), 816; b) K. J. Whitaker, P. E. Vértes, R. Romero-Garcia, F. Váša, M. Moutoussis, G. Prabhu, N. Weiskopf, M. F. Callaghan, K. Wagstyl, T. Rittman, R. Tait, C. Ooi, J. Suckling, B. Inkster, P. Fonagy, R. J. Dolan, P. B. Jones, I. M. Goodyer, E. T. Bullmore, *Proc Natl Acad Sci U S A* **2016**, *113* (32), 9105.

[4] B. T. Yeo, F. M. Krienen, J. Sepulcre, M. R. Sabuncu, D. Lashkari, M. Hollinshead, J. L. Roffman, J. W. Smoller, L. Zöllei, J. R. Polimeni, B. Fischl, H. Liu, R. L. Buckner, *J Neurophysiol* **2011**, *106* (3), 1125.
